# Interaction of Cooking-Generated Aerosols on the Human Nervous System and the Impact of Caloric Restriction Post-Exposure

**DOI:** 10.3390/nu16203525

**Published:** 2024-10-17

**Authors:** Motahareh Naseri, Sahar Sadeghi, Milad Malekipirbazari, Sholpan Nurzhan, Raikhangul Gabdrashova, Zhibek Bekezhankyzy, Reza Khanbabaie, Byron Crape, Dhawal Shah, Mehdi Amouei Torkmahalleh

**Affiliations:** 1 Department of Chemical and Materials Engineering, School of Engineering and Digital Sciences, Nazarbayev University, Astana 010000, Kazakhstan; motahareh.naseri@nu.edu.kz (M.N.);; 2 Department of Computer Science and Engineering, Chalmers University of Technology and University of Gothenburg, SE-41296 Gothenburg, Sweden; 3 Department of Biological Sciences, School of Science and Humanities, Nazarbayev University, Astana 010000, Kazakhstan; 4 Department of Chemistry, School of Engineering, Nazarbayev University, Astana 010000, Kazakhstan; 5 Department of Physics, IKK Barber School of Arts and Sciences, University of British Columbia, Kelowna, BC V1V 1V7, Canada; reza.khanbabaie@gmail.com; 6 Department of Biomedical Sciences, School of Medicine, Nazarbayev University, Astana 010000, Kazakhstan; byron.crape@nu.edu.kz; 7 Division of Environmental and Occupational Health Sciences, School of Public Health, University of Illinois at Chicago, Chicago, IL 60612, USA

**Keywords:** EEG, frying aerosols, ultrafine particles, zero calorie intake

## Abstract

**Background:** The inhalation of cooking-generated aerosols could lead to translocation to the brain and impact its function; therefore, the effects of cooking-generated aerosols on healthy adults were investigated using an electroencephalograph (EEG) during the 2 h period post-exposure. **Methods:** To explore any changes from the impact of exposure to cooking-generated aerosols on the human brain due to the absence of food intake during exposure, we divided the study participants into three groups: (A) no food intake for 2 h (2 h-zero calorie intake), (B) non-zero calorie intake, and (C) control group (simulated cooking). **Results:** The ultrafine particle concentrations increased from 9.0 × 10^3^ particles/cm^3^ at the background level to approximately 8.74 × 10^4^ particles/cm^3^ during cooking. EEGs were recorded before cooking (step 1), 60 min after cooking (step 2), 90 min after cooking (step 3), and 120 min after cooking (step 4). Comparing the non-zero calorie group with the control group, it was concluded that exposure to cooking-generated aerosols resulted in a 12.82% increase in the alpha band two hours post-exposure, compared to pre-exposure. The results revealed that zero calorie intake after exposure mitigated the impacts of cooking-generated aerosols for the alpha, beta3, theta, and delta bands, while it exacerbated effects on the whole brain for the beta1 and beta2 bands. **Conclusions:** While these are short-term studies, long-term exposure to cooking-generated ultrafine particles can be established through successive short-term exposures. These results underscore the need for further research into the health impacts of cooking-generated aerosols and the importance of implementing strategies to mitigate exposure.

## 1. Introduction

Improving indoor air quality (IAQ) is imperative as people spend approximately 65% of their time in residential environments [1,2], Several studies have shown a strong positive association between indoor air quality and health disorders such as heart disease, pulmonary disease, lung cancer, and mortality [3,4,5,6]. Activities such as cooking [7,8], smoking [8,9,10], and cleaning [11,12] are typical sources that significantly contribute to indoor air pollution. Among the different indoor activities, cooking has been identified as one of the main indoor particulate matter (PM) sources [13,14,15,16]. Kang et al. [17] conducted broiling and frying in thirty residential buildings with open-type kitchens in Seoul. The results showed a mean average PM_10_ concentration of 1269 µg/m^3^ and 475 µg/m^3^ during broiling and frying, respectively. Also, the total cooking emission rates for PM_2.5_ and PM_10_ were 0.39–20.54 mg/min and 0.58–18.47 mg/min, respectively. Klein et al. [18] conducted over 100 cooking experiments involving different oils, food types, and cooking methods. They reported that cooking causes increased emission rates of larger and unsaturated aldehydes, especially during the frying process.

While emissions of large-sized PM (>2.5 µm) have been explored in the literature, small-sized particles are often neglected. Studies on particles emitted during cooking have identified that the resulting particles can be less than 100 nm (ultrafine particles). A recent study by Shi et al. [19] found that particle mode diameters in the breathing zone (50 cm above the source) were 33 nm when heating peanut oil in the kitchen. Wallace et al. [8] also reported that the mean diameter of particles during the frying of eggs on a gas stove was 20 nm. In a similar study by Wallace et al. [8], the authors observed that the dominant size of particles during the 159 cooking tests using gas and electric stoves was 5 nm.

Ultrafine particles and particulate matter (PM) have been extensively studied for their harmful effects on the human nervous system. Fine and ultrafine particles, particularly those smaller than 2.5 microns (PM_2.5_), can penetrate the respiratory system and reach the bloodstream [20,21,22] following their deposition in the alveoli [23]. Additionally, they can access the brain by circumventing the blood-brain barrier (BBB) or through axonal transport via the olfactory nerve [24,25]. Once in the brain, these particles can induce neuroinflammation, oxidative stress, and neurodegenerative processes.

In the literature, there exist few clinical exposure studies that have addressed the neurological impact of PM resulting from smoking [26,27,28] and diesel exhaust [29] using the electroencephalogram (EEG) technique and fMRI [30]. Electroencephalography is a non-invasive and cost-effective neuroimaging technique that can measure neural oscillations on the scalp with high temporal resolution. EEG electrodes measure the electrical fields made by neurons communicating with each other. This technique has been widely used to detect neurodegenerative disorders [31,32]. However, the indoor sources of particles, including cooking, have been ignored in such studies, except in those recently published by our team [33,34]. Our previous studies [33,34] using EEG have shown that brain electrical wave patterns exhibit statistically significant changes immediately after cooking and up to 30 min post-exposure, compared to pre-exposure levels. However, the 30-min post-exposure period was too short to fully understand any effect because of the potential particle translocation to the nervous system. Furthermore, no control experiment was included in those studies to exclude the diurnal effects on brain wave patterns [33,34].

Several studies investigated the effect of fasting on brain health and function [35,36]. Intermittent fasting (IF) improved the cognitive function of a mild cognitive impairment (MCI) group compared to a control group (without IF) [37]. A reduced risk of cognitive impairment was observed in Italian adults with an eating time window duration of more than 10 h compared to those of less than 10 h [38]. People older than 70 who regularly followed fasting showed a lower risk of mental health distress [39]. Fasting has been considered a standard and effective treatment for epilepsy for many years [40]. A review indicated that fasting could also augment brain function regarding cellular stress resistance, synaptic plasticity, and neurogenesis [41]. The EEG was measured among two rat groups (one with free access to food and water ad libitum (AL) and a second group that underwent a time-restricted food (TRF) schedule). A statistically significant reduction in the power as measured by the EEG recordings during the late phase was observed in the TRF group compared to the AL-fed animals [42].

The objective of this study was to investigate the impact of exposure to cooking-generated aerosols on the human brain’s wave patterns, using EEG for up to two hours post-exposure. The second objective of this study was to explore any modifications to this impact due to zero calorie intake during exposure. While a two-hour period of zero caloric intake is not considered traditional fasting, it can still offer valuable baseline data for exploring the combined effects of fasting and exposure. This approach may help to better understand the potential synergies between brief caloric restriction and environmental factors on brain health. Notably, no prior studies have examined the impact of short-term exposure to cooking aerosols on brain function for such a brief period, making this an unexplored area of study. This gap underscores the novelty of our research, as we are the first to investigate both the direct effect of cooking aerosols on brain function and the possible synergistic interaction between zero caloric intake and short-term exposure. By bridging these two areas, our study opens up new avenues for understanding how environmental and dietary factors together influence brain health.

## 2. Materials and Methods

### 2.1. Study Participants

Study participants were recruited via advertisements placed on the university campus, including posters and emails, in accordance with the ethical guidelines approved by the university’s review board. Interested individuals were provided with comprehensive information regarding the study’s objectives and procedures prior to participation. Before the experiments, study participants were requested to complete a questionnaire (for detailed information, refer to Supplementary Material File S1) to assess their physical and mental health status and examine their cooking habits and interests. Professional chefs, pregnant women, former smokers, drug addicts, participants with cardiovascular, neurodegenerative, or respiratory diseases (e.g., asthma, bronchitis, and chronic obstructive pulmonary disease), metabolic disorders (e.g., diabetes and thyroid conditions), individuals with known allergies or sensitivities to cooking oils, people on medication that could affect respiratory, cardiovascular, or neurological function, which may interfere with the study outcomes, and individuals who had undergone recent surgeries or other major medical procedures that could impact their cardiovascular or respiratory systems were excluded from the study. Healthy participants were recruited for the experiments, based on the selection criteria outlined above, and completed the questionnaire provided in the Supplementary Materials (File S1). Eventually, 30 study participants (10 men and 20 women, with an average age of 27) were recruited for this study. A code (participant identification number) was assigned to the qualified study participants, and they were asked to sign a consent form (for detailed information, refer to Supplementary Material File S2). Nazarbayev University’s ethics committee approved the experimental procedure of this study under approval code 115/12022019, approved on 12 February 2019. Study participants were asked to have sufficient rest beforehand, avoid attending events or activities that would cause stress, anxiety, etc., avoid drinking coffee and alcoholic drinks, and take any medication before their test day. All participants had breakfast prior to the commencement of the experiment on the test day.

### 2.2. Indoor Air Quality During the Experiment

An indoor air quality monitor (IAQ, model 7545, TSI^®^, Shoreview, MN, USA) was used to measure CO_2_ concentration, indoor temperature, and humidity. DustTrak (model DRX, TSI^®^, Shoreview, MN, USA) simultaneously monitored PM_1_, PM_2.5_, PM_4_, and PM_10_ with 1-s logging intervals. A digital thermometer (Model 54IIB, Fluke^®^, [software FVF-SC2], Everett, WA, USA) measured the temperature of the food during cooking. A condensation particle counter (CPC model 3007, TSI^®^, Shoreview, MN, USA) and a NanoTracer (Philips Aerasense^®^, Eindhoven, The Netherlands) were employed to measure particle number concentrations down to 10 nm. All instruments were operated in parallel and placed close to the stove to quantify the level of exposure during cooking, except for the Nano Tracer, which was put in the living room where the study participants were present during the post-cooking period.

### 2.3. Effect Assessments

Quantitative electroencephalography (QEEG) (Discovery 24 amplifier, Brain Master^®^, Bedford, MA, USA) was employed to record the brain wave patterns. The device has 24 channels and a sampling rate of 256 Hz. We employed an electro-cap with 19 active electrodes placed according to the 10–20 international system (Fp1, Fp2, F3, F4, C3, C4, P3, P4, O1, O2, F7, F8, T3, T4, T5, T6, Fz, Cz, and Pz), with linked-ear montage. The Fz node was used as the ground electrode and was placed on the electro cap. The online 0.1–40 Hz band pass filter was applied. Visual selection was initially used to avoid eye blinking and muscle and cardiac artifacts. On average, 34 s of artifact-free signals were selected for the analysis. The EEG data were processed offline by MATLAB (R 2020b) for six frequency bands, including delta (1–3.5 Hz), theta (4–7.5 Hz), alpha (8–12 Hz), beta1 (12–15 Hz), beta2 (15–17.5 Hz), and beta3 (18–25 Hz). The electrodes’ relative power was collected for each subject and each session of EEG recording. Among the five lobes of the brain, the activities of the frontal (right and left), temporal (right and left), central, parietal, and occipital lobes were measured using F3, F4, F7, F8, Fp1, FP2, Fz; T3, T4, T5, T6; Cz, C3, C4; P3, P4, Pz; and O1 and O2, respectively.

### 2.4. Experimental Design

The experiments were conducted in a one-bedroom apartment at Nazarbayev University with an electric stove (Figure S1). The volume of the apartment was approximately 115 m^3^. One hundred grams of low-fat ground beef was used to prepare three pan kebabs. The meat was mixed with 1 g salt, 1 g pepper, 1 g turmeric, and 20 g shredded onion. The mixture was divided into three parts to make three pan kebabs, each weighing 40 g. A 20-centimeter aluminum pan with PTFE coating was preheated (medium heat setting (level 6)), and, after two minutes, 25 mL of sunflower oil was added to the pan. Six minutes after heating the oil, three pieces of pan kebab were placed into the pan. The pan kebabs were flipped using a wooden spatula at 11, 14, and 17 min. The stove was switched off at minute 20 while particle monitoring continued until the concentrations returned to the background level. During the cooking process, the kitchen hood was off. All windows and doors of the experimental apartment were closed during the experiment to minimize the indoor penetration of ambient gases and particles. This study was designed to evaluate how these everyday activities, combined with exposure, contribute to the overall effects. The diurnal effect, which includes the impact of normal daily routines like eating, was a key component of our analysis. By allowing participants to follow their usual eating patterns, we accounted for the way in which realistic lifestyle factors might interact with exposure to cooking emissions.

Study participants were divided into two groups for exposure experiments; one group was instructed to eat food (non-zero calorie intake group) if needed, while the other was restrained and asked to undergo 2 h of non-intake of food during the experiments (2 h-zero calorie intake). The participants in the non-zero calorie intake group and the 2 h-zero calorie intake group were different individuals; there was no overlap between the two groups. Study participants were asked to sit and rest for 30 min to adapt after entering the apartment. After that, the first EEG was recorded as a background measurement. Next, as noted above, the study participants stood beside the stove and fried the pan kebab using the experimental protocol. After cooking, the study participants sat on the sofa for two hours. The second, third, and fourth EEGs were recorded 60, 90, and 120 min after cooking. To reduce any electrical noise during EEG measurement, all electrical appliances were unplugged, and all necessary devices for EEG recording and particle counters were operated under battery power.

### 2.5. Control Experiments

Eight study participants in the exposure study participated in the control experiments, with their involvement specified as follows: three participants were part of the non-zero calorie intake group, and five participants were part of the two-hour zero calorie intake group. During the control experiments, study participants performed the same activities as the exposure experiments; however, the stove was turned off to avoid any emissions. The participants rested for 30 min after their arrival for adaptation and stayed at the apartment for 3 h. They were allowed to eat food if needed, like the non-zero calorie intake group in the exposure experiments. They were asked to stand next to the stove during cooking, but the stove was off (simulated cooking). No ventilation was used during the simulated cooking, and the doors and windows were closed during the experiments. The brain EEGs of the control study participants were measured at the same time intervals as the exposure group (background level and 60, 90, and 120 min after simulated cooking). The study participants were sitting on the sofa during the post-exposure experiments.

### 2.6. Brain Wave Pattern Percentage Calculation

The participant’s relative power of the control, non-zero calorie intake, and 2 h-zero calorie intake groups in each lobe were averaged to identify diurnal effect percentage, diurnal, exposure, and zero calorie effects. The percentage between the relative power of step 1 (before cooking) and step 4 (120 min after cooking) was calculated. The percentage of exposure effect was calculated by subtracting the non-zero calorie intake group percentage (diurnal and exposure effects) from the control group percentage (general effect). The effect of zero calorie intake on exposure was established by subtracting the 2 h-zero calorie intake group percentage (diurnal and exposure and zero calorie effect) from the non-zero calorie intake group (diurnal and exposure effects percentage). If the exposure changed the brain in a particular direction (increase or decrease) and the zero calorie intake reduced that effect, the term mitigate was used. Still, if the zero calorie intake effect was in the same direction as the exposure and worsened it, the term exacerbating effect was used.

### 2.7. Statistical Analysis Method

Relative power for all electrodes was compared among the sessions (pre- and post-exposure). The non-parametric Friedman test [43] was implemented using R software (version 4.4.0) to measure the same characteristics for each subject at different intervals (pre-exposure, 60 min, 90 min, and 120 min post-exposure) because the relative power data failed the Shapiro–Wilks normality test. The Friedman test detects the differences across multiple test attempts. The null hypothesis (H_0_) for the Friedman test is that there are no differences among the population’s distribution of scores. The alternative hypothesis shows that at least one related population’s distribution of scores is different from the others. In cases where the Friedman test showed a statistically significant difference between pre-and post-exposure results, the Wilcoxon test as a post hoc test [44] was used to compare two paired groups (among the data available for pre-exposure, 60 min, 90 min, and 120 min post-exposure), where:

H_0_ = µ_1_ = µ_2_ = µ_3_= µ_4_;
H_0_ = mean RP is the same at all times;
H_1_ = mean RP is significantly different at one or more time points;
µ is the population mean of RP, and the related groups are the subjects before, during, and after cooking.

## 3. Results

### 3.1. Indoor Air Quality During the Experiment

**Figure 1:**
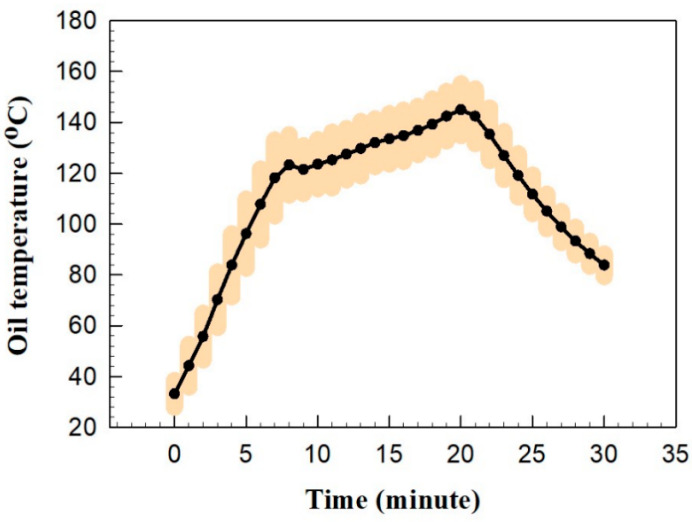
Oil temperature variations with time during frying, along with the range obtained from different runs.

Figure 1 shows the oil temperature during the frying of pan kebabs. The first two minutes recorded the empty pan temperature. The oil temperature reached 70 °C one minute after adding it to the heated pan. The oil temperature generally showed an increasing trend and reached a maximum temperature of 145 °C, maintaining this until the stove was turned off at minute 20. A slight drop in the oil temperature at minute 8 corresponded to adding the pan kebabs to the pan. The pan kebabs were kept at room temperature before starting the cooking process. After turning off the stove, the oil temperature decreased sharply.

**Figure 2:**
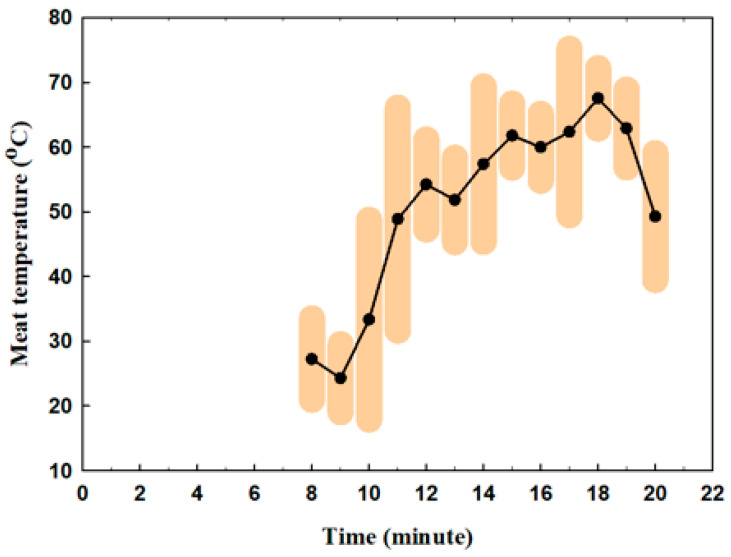
The meat temperature variations with time during frying, within the highlighted range of temperature, among the different runs.

Figure 2 presents the meat temperature according to time during frying. The meat was added to the pan at minute 8. Its temperature increased from 27 °C to 67.5 °C by minute 17. The reductions in temperature at minutes 11, 15, and 17 correspond to the flipping of the meat inside the pan.

**Figure 3:**
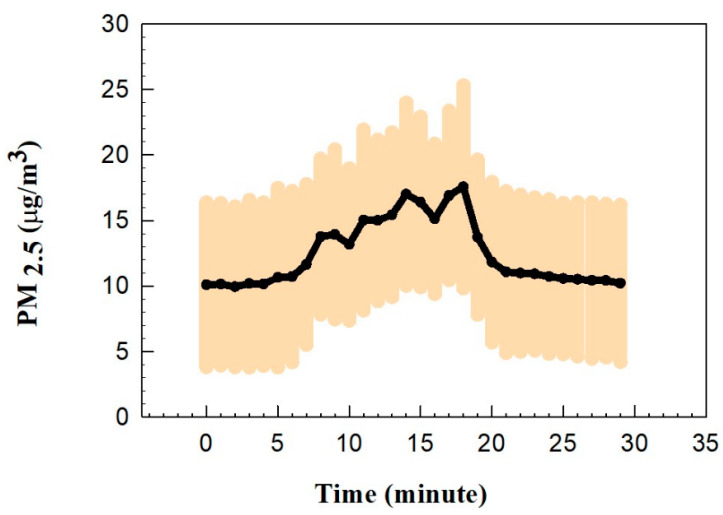
The PM_2.5_ concentration variations with time during cooking.

Figure 3 shows the average PM_2.5_ concentrations during the frying of pan kebabs. The PM_2.5_ concentration showed no changes during the first two minutes of heating the empty pan, which is consistent with previous findings [45,46]. The PM concentration increased from the background level of approximately 1.0 µg/m^3^ to a maximum of 17.0 µg/m^3^ at minute 18. The fluctuations and decreases in PM after minutes 11, 14, and 17 could be related to the flipping over of the pan kebabs inside the pan. Buonanno et al. [47] and Amouei Torkmahalleh et al. [14] reported that heating cooking oil produces more particles than when heating meat. Thus, PM concentration fluctuations during flipping time could be due to replacing the oil surface with the meat surface. The stove was turned off at minute 20, and the PM concentration declined to a background level approximately 10 min after the end of cooking.

**Figure 4:**
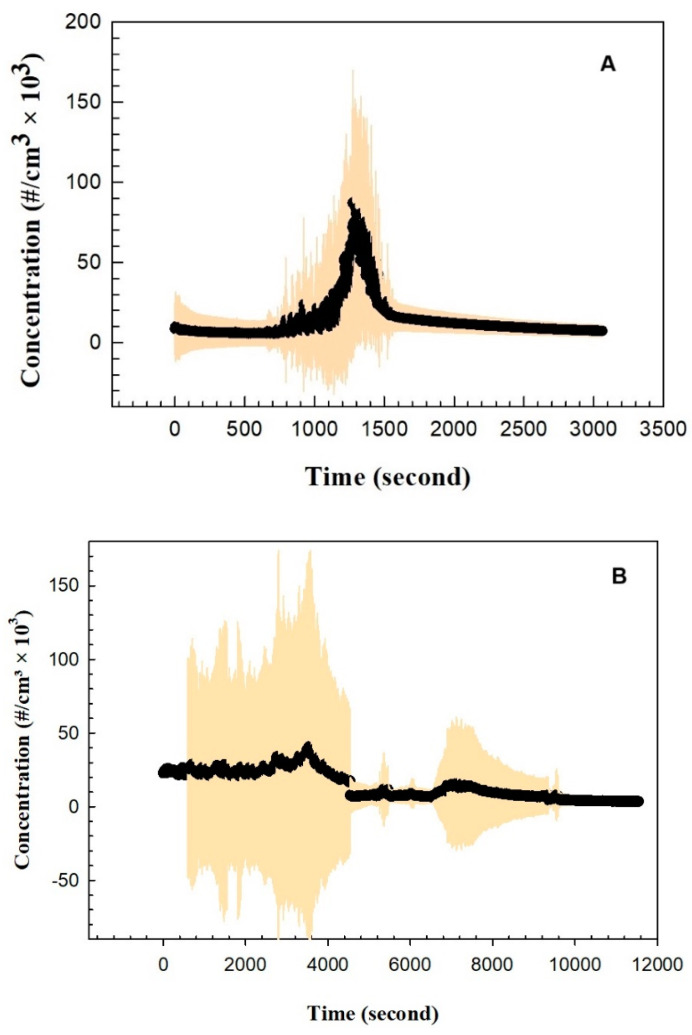
Particle number concentrations: variations with time during frying, measured next to the stove, recorded using CPC (**A**) and during the post-frying period, measured next to the participant, recorded using NanoTracer (**B**).

Figure 4 shows the particle concentrations as measured next to the electric stove and near the study participants, respectively. The particle number concentration during cooking increased from 9.0 × 10^3^ particles/cm^3^ to the maximum of 8.74 × 10^4^ particles/cm^3^ at minute 20 and dropped after the gas was switched off. The maximum particle number concentration measured next to the participants was observed at the end of the cooking to be approximately 4.04 × 10^4^ particles/cm^3^. The average particle number concentration during post-exposure, measured next to the participants, was approximately 7.5 × 10^3^ particles/cm^3^. The data for CO_2_ concentration, relative humidity (RH), and ambient temperature during the cooking time, including detailed graphs, are provided in the Supplementary Materials (Figures S2 and S3).

### 3.2. Brain Response

#### 3.2.1. Control Experiments Results

**Figure 5:**
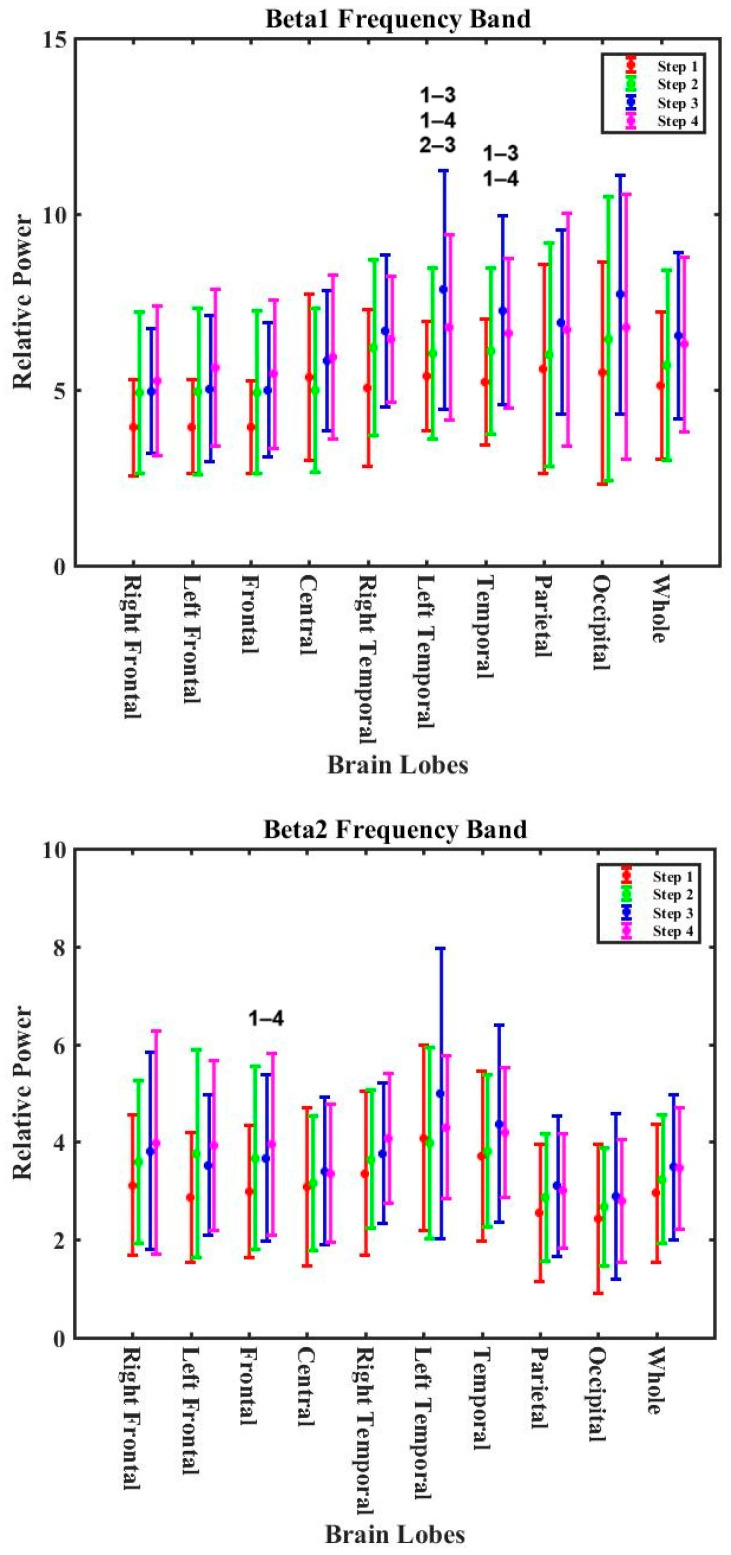
The variations in the beta1 and beta2 bands for the control experiment for different lobes of the brain (the numbers on the bar graph indicate the stages in which there was a statistically significant difference in relative power).

Table S1 shows the *p*-values for all bands at all lobes during the control experiments for four different steps, including before cooking (step 1), 60 min after cooking (step 2), 90 min after cooking (step 3), and 120 min after cooking (step 4). Different trending for the bands was observed when comparing the bands’ results after 2 h post-exposure with the background level (Step 1). Beta1, beta2, beta3, and theta values showed an increasing trend; however, delta and alpha decreased after two hours. These changes were only statistically significant in beta1 and beta2 (Figure 5). The relative power in the beta1 band statistically significantly increased by approximately 26.0% for the left temporal lobe (RP_step1_ = 5.40 ± 0.94 with RP_step3_ = 7.84 ± 1.47 and RP_step4_ = 6.65 ± 1.13) and temporal lobe (RP_step1_ = 5.23 ± 1.27 with RP_step3_ = 7.26 ± 1.60 and RP_step4_ = 6.61 ± 1.48). The relative power in the beta2 band statistically significantly increased in the frontal lobe (RP_step1_ = 2.97 ± 0.46 with RP_step3_ = 3.62 ± 0.46 and RP_step4_ = 3.83 ± 0.63). Our findings show that an individual’s EEG reflects diurnal effects that are in agreement with the literature. Studies demonstrated the effect of the diurnal variation on the EEG signal power [48,49,50]. Cummings et al. [51] reported + 20% changes in the mean actual frequency waveband power of the EEGs of male and female groups over 24 h due to biological circadian variations.

#### 3.2.2. Electrode Analysis for Exposure Experiments

Tables S2 and S3 show the *p*-values calculated after comparing the RP values across different post-exposure steps, registered for all electrodes and frequency bands among the two groups (2 h-zero calorie intake and non-zero calorie intake groups). For the non-zero calorie intake group, no statistically significant differences were found between the delta and theta bands across all electrodes. The alpha band showed statistically significant increases in steps 1–3 for T3. The beta1 band showed a statistically significant reduction in steps 2–4 and 3–4 for FP2 and F8 and in steps 3–4 for F3, Fz, and Cz. The beta2 band showed a statistically significant reduction between steps 2–4 and 3–4 for F8 and in steps 3–4 for T3. The beta3 band showed a statistically significant decrease between steps 2–4 and 3–4 for FP2, an increase between steps 1–2 and 1–3, a reduction between steps 2–4 and 3–4 for F8, increases between steps 1–3 and 2–3, a reduction in steps 3–4 for Cz and P3, increases between steps 1–2 and 1–3 for Pz, an increase in steps 2–3 and a reduction in steps 3–4 for T5. The relative power changes for alpha, beta1, beta2, and beta3 in other electrodes were insignificant.

For the 2 h-zero calorie intake group, no statistical changes were observed in beta1 and beta2; however, beta3 only showed a statistically significant difference in O2. The delta band showed statistically significant changes between steps 1–3 (P3, P4, and O2), steps 2–3 (P3, P4, T6, and O2), and steps 3–4 (P3 and O2). The theta values showed significant changes in electrodes P3 (steps 2–3 and 3–4) and Pz (steps 2–3). The alpha band alternated significantly for P3 (steps 2–3 and 3–4) and T6 (steps 2–3 and 2–4).

#### 3.2.3. Lobe Analysis for Exposure Experiments

**Figure 6:**
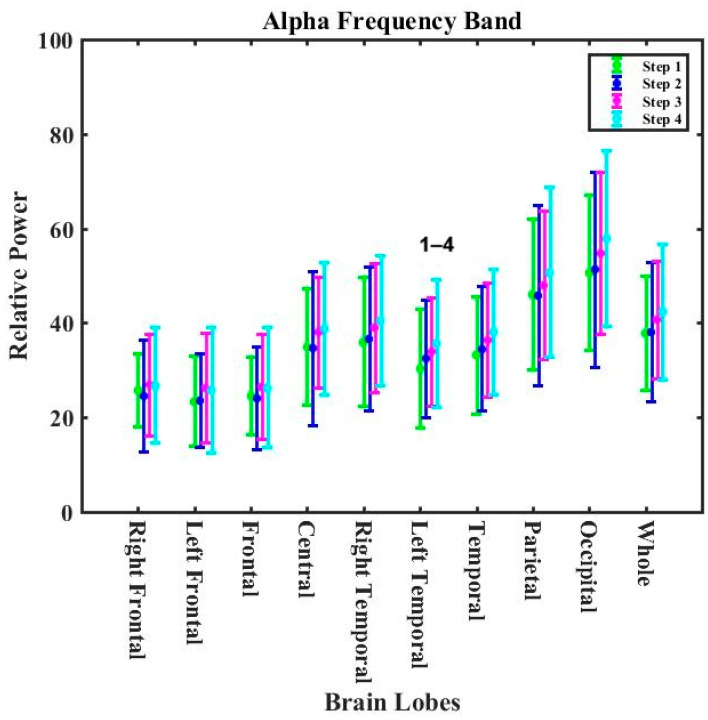
The variations in the alpha band for different exposure periods for different lobes of the brain (non-zero calorie intake group). The numbers on the bar graph indicate the stages in which there was a statistically significant difference in relative power.

Tables S4 and S5 present the *p*-values for all bands and all lobes during the experiments for the non-zero calorie intake group and 2 h-zero calorie intake group. Tables S4 and S5 show that the alpha, beta1, beta2, and beta3 bands statistically significantly differed during the exposure in the non-zero calorie intake group. No statistically significant changes were observed for the theta and delta bands in the non-zero calorie intake group. An increasing trend for the alpha band was observed in all lobes of the whole brain (Figure 6). The relative power of the alpha band statistically significantly increased (13.6%) for the left temporal lobe after 2 h post-cooking (RP_step1_= 31.3 ± 9.8 with RP_step4_ = 35.6 ± 13.4).

**Figure 7:**
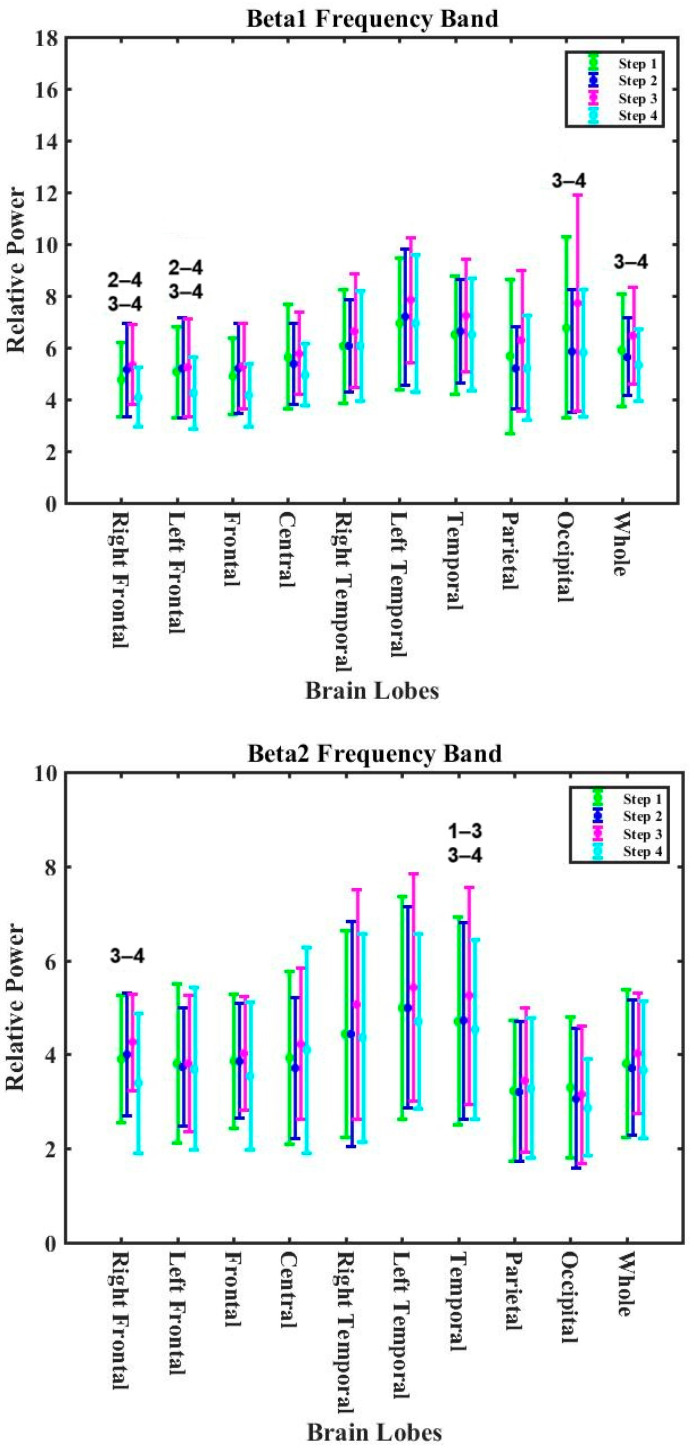

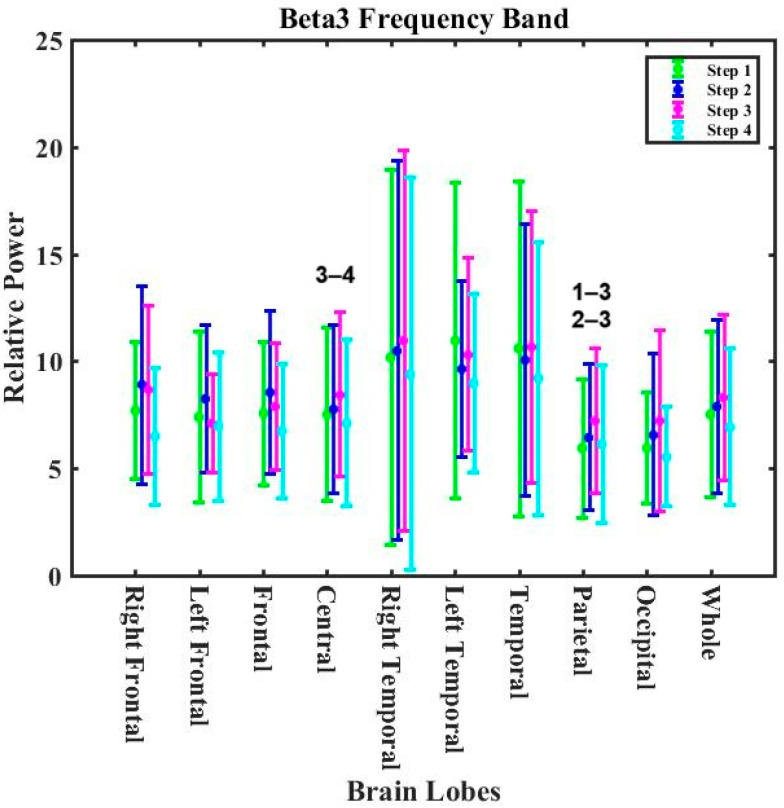
The variations in the beta1, beta2, and beta3 bands for different exposure periods in the different lobes of the brain (non-zero calorie intake group). The numbers on the bar graph indicate the stages in which there was a statistically significant difference in relative power.

The decreasing trends for the beta1, beta2, and beta3 bands were observed in the whole of the brain (Figure 7). The relative power in the beta1 band was statistically significant for the right frontal (RP_step2_ = 5.15 ± 1.79 and RP_step3_ = 5.41 ± 1.48 compared to RP_step4_ = 4.12 ± 1.19), frontal (RP_step2_ = 5.21 ± 1.72 and RP_step3_ = 5.33 ± 1.60 with RP_step4_ = 4.22 ± 1.29) and occipital (RP_step3_ = 7.40 ± 3.88 with RP_step4_ = 5.91 ± 2.64) lobes. The relative power in the beta2 band was statistically significant for the temporal (RP_step1_ = 4.93 ± 5.60 with RP_step3_ = 4.76 ± 2.75) and right frontal (RP_step3_ = 4.22 ± 1.05 with RP_step4_ = 3.38 ± 1.47) lobes. The relative power in the beta3 band statistically significantly decreased for the central lobe (RP_step3_ = 8.46 ± 3.85 with RP_step4_ = 7.07 ± 3.79). The relative power in the beta3 band increased significantly in the parietal lobe (RP_step1_ = 5.87 ± 3.43 and RP_step2_ = 6.48 ± 3.43 with RP_step3_ = 7.08 ± 3.32).

**Figure 8:**
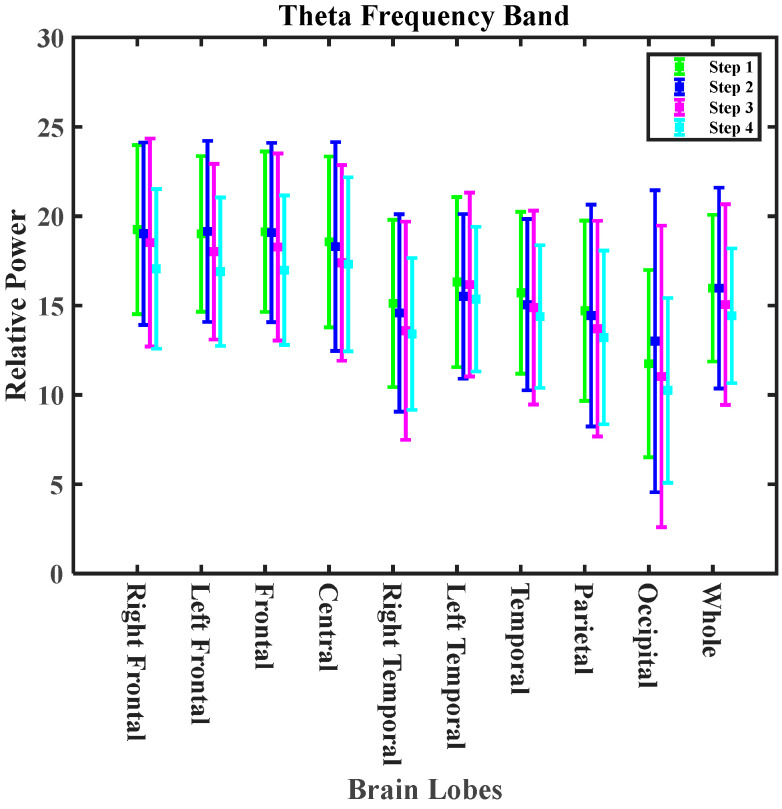

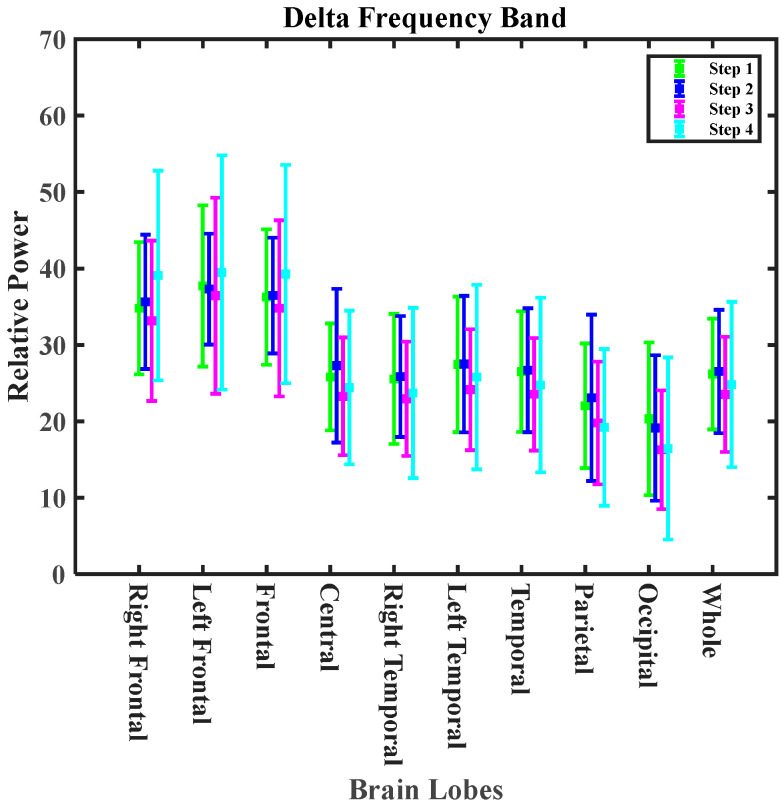
The variations in the delta and theta bands for different exposure periods for the different lobes of the brain (non-zero calorie intake group).

Figure 8 shows the oscillation pattern in the delta and theta bands during cooking experiments in the non-zero calorie intake group. The theta and delta bands showed a decreasing pattern in the whole brain. A decreasing pattern in the delta band is consistent with the observed results during exposure to cooking-generated aerosols from meat frying on an electric stove [34] and exposure to cooking-generated aerosols from chicken frying on a gas stove [33]. However, the observed changes in EEG at the different steps of exposure could be attributed to exposure and diurnal effects. The observed changes in EEG frequency bands among the zero calorie intake group are shown in Figure S4.

## 4. Discussion

Our results showed the coexistence of three factors that impacted EEG: exposure to cooking-generated aerosols, zero calorie intake, and diurnal effects. A further analysis was conducted based on two key assumptions, to separate the effect of each factor. Firstly, it was assumed that the diurnal effects in terms of the percentage of change observed during different times of the day in the control group were the same as in other exposed groups. Secondly, it was assumed that the exposure effects were similar for the zero calorie intake and non-zero calorie intake groups. The results of this analysis are presented in Table 1.

Table 1 shows the percentage of changes in the different frequency bands after 2 h post-exposure compared to the values before exposure (background of each group) for the control, non-zero calorie intake, and 2 h-zero calorie intake groups. The percentage of the changes has been deducted from the changes observed in the control group, to exclude the diurnal effects. For example, Table 1 shows that the alpha band underwent a 2.54% decrease in the whole brain 2 h after the simulated cooking time. In contrast, the alpha band experienced a 10.28% increase 2 h post-exposure in the whole brain compared to the background values for the non-zero calorie intake group, which is attributed to both diurnal and exposure effects. Since the 2 h post-exposure in exposed groups and two hours after arrival in control groups are at the same time of the day and, likewise, the time before exposure in the exposed groups and arrival time in the control group is identical, then the diurnal effects could be excluded by subtracting the 2.54% decreases from the 10.28% increases. Thus, it is concluded that exposure to cooking-generated aerosols resulted in 12.82% increases in the alpha band after 2 h post-exposure compared to the values before exposure. However, when the percentage of the changes was compared among the two exposed groups, it was found that the 12.82% increase due to exposure was reduced by 8.15% due to zero calorie intake. Thus, zero calorie intake tended to significantly mitigate the effect of cooking-generated aerosol exposure on the whole brain’s alpha band.

Table 1 presents the actual effect of exposure and zero calorie intake on the whole brain. It was found that exposure to cooking-generated aerosols decreased all beta bands and increased the delta band within 2 h post-exposure on the whole brain. This effect is consistent with the changes to the beta and delta bands of the brain of dementia patients [52,53,54,55,56,57]. However, this consistency was not found for the theta and alpha bands. Zero calorie intake showed an interesting impact on the brain. Table 1 shows that zero calorie intake mitigated the effects of exposure to cooking-generated aerosols on the whole brain for the alpha, beta3, delta, and theta bands while it exacerbated the effects on the beta1 and beta2 bands. Tables S6–S10 show similar analyses for the different lobes of the brain. The mitigating effects of zero calorie intake after exposure to cooking-generated aerosols for the alpha and beta1 bands were observed in all lobes except the occipital lobe, which showed an exacerbating effect. The mitigating effect of zero calorie intake was observed for the beta2 band in the central and parietal lobes and for the beta3 band in the central and frontal lobes. Exacerbating effects were observed in the other lobes for the beta2 and beta3 bands. Zero calorie intake mitigated the effects of exposure to cooking-generated aerosols in the whole lobes of the brain except in the theta bands; the whole lobes underwent the mitigating effect of zero calorie intake, except for the frontal and occipital lobes concerning the delta band. However, a study on eight healthy volunteers during fasting during Ramadan and during non-fasting periods showed no difference in the EEG absolute power in the delta, theta, alpha, and beta frequency bands [58].

The mechanism of the effects of PM and UFP on the body depends on their solubility, origin, composition, and ability to produce reactive oxygen [59]. Toxicological studies demonstrated that olfactory and blood circulation are the two main pathways for the translocation of particles to the brain. The olfactory route starts from nasal deposition and reaches the central nervous system (CNS) through the olfactory bulb in the frontal lobe [60,61]. The dendrites of olfactory receptor neurons are in direct contact with the environment and run from the sensory epithelium to the olfactory bulb. Therefore, the most likely routes of access to the CNS for UFPs and toxins are pinocytosis and neuronal transport [62]. The respiratory tract system creates a systemic route for particle translocation [63].

Several studies have shown that fasting can improve brain function and can be used as a disease treatment method. The mechanism of food restriction slowing age-related changes in the brain is not established but may involve reduced mitochondrial oxyradical production [64,65] and decreased levels of cellular oxidative stress [66]. The study’s results on 99 elderly subjects with MCI (mild cognitive impairment) showed a decrease in the markers of oxidative stress in the IF group compared to the non-IF group. The malondialdehyde (MDA) level in the IF group decreased after 36 months, while the mean MDA in the non-IF group increased over time [37]. Also, an analysis of the inflammatory markers showed a significant decrease in C-reactive protein (CRP) among the IF group compared to the non-IF group.

Fasting could mitigate aging, a significant risk factor in cognitive impairment, dementia, and neurological disease. A deterioration in the extent of dendritic branching in the hippocampus and in the superficial cortical layer of the prefrontal cortex occurs as a result of aging. In contrast, aged neurons increase the density of calcium channels and after-hyperpolarization (AHP) potential, coinciding with a reduction in brain-derived neurotrophic factor (BDNF) levels. A decrease in BDNF levels has been observed to correspond with age-related cognitive deficits [38]. Many studies have indicated that intermittent fasting could potentially boost synaptic plasticity, neurogenesis, and neuroprotective mechanisms, mainly by increasing BDNF [67,68]. The enhanced production of BDNF has been reported as a crucial neurological adaptation to IF [69]. Also, in many rodent studies, IF triggered the expression of BDNF and increased BDNF brain levels [70,71,72]. Another physiological mechanism of IF involves increasing the expression of synaptic proteins in the brain during aging, which is associated with increased inflammation and oxidative stress [73,74] and also improved mitochondrial respiratory activity [75].

While a two-hour period of zero caloric intake is not considered traditional fasting, it still provides valuable baseline data for exploring the combined effects of caloric restriction and environmental exposure on brain health. Short periods of zero caloric intake could temporarily alter the metabolic processes, affecting glucose levels, hormonal responses, and even brain activity. These metabolic shifts, although not as profound as those seen in extended fasting, may influence how the brain responds to external stressors, such as particulate matter (PM) from cooking aerosols. By utilizing this short zero-calorie period as a baseline, researchers can begin to explore how longer fasting periods, combined with environmental factors like air pollution, might interact to affect brain health. These baseline data serve as an essential step in developing a deeper understanding of how fasting and environmental exposure together influence neurological outcomes, potentially leading to new strategies for minimizing the harmful effects of aerosols on the brain.

## 5. Limitations of This Study and Future Work

This study utilized a different sample of human subjects for the control group compared to the two exposed groups and assumed that the diurnal effect observed in the control group could be extended to other exposed groups (zero calorie intake and non-zero calorie intake groups). A study employing the same human subject sample for both exposed and control groups would eliminate the diurnal effect and other confounders.

While we aimed to minimize bias through a rigorous participant selection process, there may still be inherent biases due to self-reported data on health status and cooking habits. This reliance on subjective measures can introduce variability and affect the accuracy of the findings.

The results of the present study are based on a relatively small sample size for each exposed group, as well as the control group. A power analysis based on the results of the current study is needed to determine the appropriate sample size for future studies.

The present study examined the post-exposure effects for up to 2 h. Extending the post-exposure effects to 24 h would provide a better insight into the kinetics of the potential for particle translocation to the nervous system.

We aimed to simulate real-world conditions wherein participants followed their usual eating routines; the study did not include separate control groups that were specifically designed to isolate the effects of different caloric intakes on EEG patterns. This decision was based on the assumption that normal eating patterns, neither high nor low in calories, serve as a reference point in daily-life scenarios. However, future studies incorporating additional control groups could provide a clearer understanding of how specific eating patterns independently influence EEG readings, distinct from the effects of exposure to environmental factors such as cooking emissions.

## 6. Conclusions

In our study, we investigated the effects of cooking-generated aerosols on brain activity as measured by EEG monitoring, emphasizing the interactions between exposure to these aerosols, zero calorie intake, and diurnal variations. Our findings indicate that exposure to cooking-generated aerosols significantly impacts brain function, as evidenced by a notable reduction in the theta and all beta frequency bands after two hours post-exposure. Conversely, we observed increases in the alpha and delta bands, highlighting the complex neurophysiological responses elicited by inhaling ultrafine particles.

Importantly, the introduction of zero calorie intake was found to mitigate some of these adverse effects. Specifically, zero calorie intake reduced the impact of cooking-generated aerosols on the alpha, beta3, theta, and delta bands, suggesting a protective mechanism that may be attributed to altered metabolic processes during periods of caloric restriction. However, it is critical to note that zero calorie intake also exacerbated the effects on the beta1 and beta2 bands, indicating a nuanced relationship between fasting and environmental exposure.

These results underscore the need for further exploration into the multifactorial nature of brain responses to environmental pollutants, particularly on how dietary habits may influence the neurological impacts of such exposures. Our study lays the groundwork for future research to investigate the long-term effects of and potential strategies for minimizing the harmful consequences of cooking-generated aerosols on brain health, particularly in vulnerable populations. As we continue to grapple with the implications of air quality and nutrition and their effect on cognitive function, it becomes increasingly imperative to consider the intricate interplay between these factors in public health initiatives and personal health practices.

## Figures and Tables

**Table 1:** The different brain wave patterns recorded in the whole of the brain after two hours. Percentage values refer to the percentage of the relative power changes after two hours, compared to the background level.

Frequency Band	Control Group (Diurnal Effect)	Non-Zero Calorie Intake Group (Diurnal and Exposure Effects)	Effect of Exposure	2 h-Zero Calorie Intake Group (Diurnal and Exposure and Zero Calorie Intake Effects)	Effect of Zero Calorie Intake on the Exposure Effect
Alpha	decreasing trend 2.54%	increasing trend 10.28%	12.82% increases	increasing trend 2.13%	8.15% mitigating effect
Beta1	increasing trend 26.24%	decreasing trend 3.10%	29.34% reductions	decreasing trend 4.63%	1.53% exacerbating effect
Beta2	increasing trend 17.80%	decreasing trend 1.69%	19.49% reductions	decreasing trend 4.47%	2.78% exacerbating effect
Beta3	increasing trend 12.73%	decreasing trend 5.80%	18.53% reductions	decreasing trend 4.28%	1.52% mitigating effect
Delta	decreasing trend 12.49%	decreasing trend 7.69%	6.40% increases	decreasing trend 6.40%	1.29% mitigating effect
Theta	increasing trend 0.31%	decreasing trend 4.98%	5.29% reductions	increasing trend 7.79%	12.77% mitigating effect

## Data Availability

The data presented in this study are available on request from the corresponding author due to privacy and ethical concerns.

## Supplementary Materials

The following supporting information can be downloaded at: https://www.mdpi.com/article/10.3390/nu16203525/s1, Figure S1: The test room setup; Figure S2: CO_2_ concentration during cooking time; Figure S3: Room temperature and relative humidity during cooking time; Figure S4. The variations in the delta and theta bands for different exposure periods for different lobes of the brain (2 h-zero calorie group); File S1: The questionnaire form; File S2: The consent form; Table S1: The *p*-values for all bands in all lobes during the control experiments for four different steps, including before cooking (step 1), 60 min after cooking (step 2), 90 min after cooking (step 3), and 120 min after cooking (step 4).; Table S2: The *p*-values calculated from comparing the RP values across the different post-exposure steps, registered for all electrodes and frequency bands among the 2 h-zero calorie intake group; Table S3: The *p*-values calculated from comparing the RP values across different post-exposure steps, registered for all electrodes and frequency bands among the non-zero calorie intake group; Table S4: The *p*-values for all bands at all lobes during the experiments for the 2 h-zero calorie intake group; Table S5: The *p*-values for all bands at all lobes during the experiments for the non-zero calorie group; Table S6. Different brain wave patterns are recorded in the brain’s central lobe after two hours; Table S7: Different brain wave patterns recorded in the frontal lobe of the brain after two hours; Table S8: Different brain wave patterns recorded in the occipital lobe of the brain after two hours; Table S9: Different brain wave patterns recorded in the parietal lobe of the brain after two hours; Table S10: Different brain wave patterns recorded in the temporal lobe of the brain after two hours.

## References

[1] Baeza_Romero M.T., Dudzinska M.R., Torkmahalleh M.A., Barros N., Coggins A.M., Ruzgar D.G., Kildsgaard I., Naseri M., Rong L., Saffell J. (2022). A review of critical residential buildings parameters and activities when investigating indoor air quality and pollutants. Indoor Air.

[2] Brasche S., Bischof W. (2005). Daily time spent indoors in German homes–baseline data for the assessment of indoor ex-posure of German occupants. Int. J. Hyg. Environ. Health.

[3] Badida P., Krishnamurthy A., Jayaprakash J. (2023). Meta analysis of health effects of ambient air pollution exposure in low-and middle-income countries. Environ. Res..

[4] Bălă G.-P., Râjnoveanu R.-M., Tudorache E., Motișan R., Oancea C. (2021). Air pollution exposure—The (in)visible risk factor for respiratory diseases. Environ. Sci. Pollut. Res..

[5] Chen W., Wang X., Chen J., You C., Ma L., Zhang W., Li D. (2023). Household air pollution, adherence to a healthy lifestyle, and risk of cardiometabolic multimorbidity: Results from the China health and retirement longitudinal study. Sci. Total Environ..

[6] Kumar P., Singh A.B., Arora T., Singh S., Singh R. (2023). Critical review on emerging health effects associated with the indoor air quality and its sustainable management. Sci. Total Environ..

[7] Wallace L. (2006). Indoor sources of ultrafine and accumulation mode particles: Size distributions, size-resolved concentrations, and source strengths. Aerosol Sci. Technol..

[8] Wallace L., Wang F., Howard-Reed C., Persily A. (2008). Contribution of gas and electric stoves to residential ultrafine particle concentrations between 2 and 64 nm: Size distributions and emission and coagulation rates. Environ. Sci. Technol..

[9] Brauer M., Hirtle R., Lang B., Ott W. (2000). Assessment of indoor fine aerosol contributions from environmental tobacco smoke and cooking with a portable nephelometer. J. Expo. Sci. Environ. Epidemiol..

[10] DeCarlo P.F., Avery A.M., Waring M.S. (2018). Thirdhand smoke uptake to aerosol particles in the indoor environment. Sci. Adv..

[11] Singer B.C., Coleman B.K., Destaillats H., Hodgson A.T., Lunden M.M., Weschler C.J., Nazaroff W.W. (2006). Indoor secondary pollutants from cleaning product and air freshener use in the presence of ozone. Atmos. Environ..

[12] Wong J.P.S., Carslaw N., Zhao R., Zhou S., Abbatt J.P.D. (2017). Observations and impacts of bleach washing on indoor chlorine chemistry. Indoor Air.

[13] Abdullahi K.L., Delgado-Saborit J.M., Harrison R.M. (2013). Emissions and indoor concentrations of particulate matter and its specific chemical components from fast: A review. Atmos. Environ..

[14] Torkmahalleh M.A., Gorjinezhad S., Unluevcek H.S., Hopke P.K. (2017). Review of factors impacting emission/concentration of cooking generated par-ticulate matter. Sci. Total Environ..

[15] Schiavon M., Rada E.C., Ragazzi M., Antognoni S., Zanoni S. (2015). Domestic activities and PM generation: A contribution to the understanding of indoor sources of air pollution. Int. J. Sustain. Dev. Plan..

[16] Zhao Y., Liu L., Tao P., Zhang B., Huan C., Zhang X., Wang M. (2019). Review of effluents and health effects of cooking and the performance of kitchen ventilation. Aerosol Air Qual. Res..

[17] Kang K., Kim H., Kim D.D., Lee Y.G., Kim T. (2019). Characteristics of cooking-generated PM10 and PM2.5 in residential buildings with different cooking and ventilation types. Sci. Total Environ..

[18] Klein F., Baltensperger U., Prévôt A.S.H., El Haddad I. (2019). Quantification of the impact of cooking processes on indoor concentrations of volatile organic species and primary and secondary organic aerosols. Indoor Air.

[19] Shi L., Liu Z., Wen W., Son J.H., Li L., Wang L., Chen J. (2023). Spatial distributions of particle number size distributions generated during cooking processes and the impacts of range hoods. Sci. Total Environ..

[20] Allen J.L., Oberdorster G., Morris-Schaffer K., Wong C., Klocke C., Sobolewski M., Conrad K., Mayer-Proschel M., Cory-Slechta D.A. (2017). Developmental neurotoxicity of inhaled ambient ultrafine particle air pollution: Parallels with neuropathological and behavioral features of autism and other neurodevelopmental disorders. Neurotoxicology.

[21] Bhargava A., Tamrakar S., Aglawe A., Lad H., Srivastava R.K., Mishra D.K., Tiwari R., Chaudhury K., Goryacheva I.Y., Mishra P.K. (2018). Ultrafine particulate matter impairs mitochondrial redox homeostasis and activates phosphatidylinositol 3-kinase mediated DNA damage responses in lymphocytes. Environ. Pollut..

[22] Clifford S., Mazaheri M., Salimi F., Ezz W.N., Yeganeh B., Low-Choy S., Walker K., Mengersen K., Marks G.B., Morawska L. (2018). Effects of exposure to ambient ultrafine particles on respiratory health and systemic inflammation in children. Environ. Int..

[23] Guo L., Johnson G.R., Hofmann W., Wang H., Morawska L. (2020). Deposition of ambient ultrafine particles in the respiratory tract of children: A novel experimental method and its application. J. Aerosol Sci..

[24] Kreyling W.G. (2016). Discovery of unique and ENM—Specific pathophysiologic pathways: Comparison of the translocation of inhaled iridium nanoparticles from nasal epithelium versus alveolar epithelium towards the brain of rats. Toxicol. Appl. Pharmacol..

[25] Simko M., Mattsson M.-O. (2014). Interactions between nanosized materials and the brain. Curr. Med. Chem..

[26] Domino E.F., Ni L., Thompson M., Zhang H., Shikata H., Fukai H., Sakaki T., Ohya I. (2009). Tobacco smoking produces widespread dominant brain wave alpha frequency increases. Int. J. Psychophysiol..

[27] Golding J. (1988). Effects of cigarette smoking on resting EEG, visual evoked potentials and photic driving. Pharmacol. Biochem. Behav..

[28] Rass O., Ahn W.-Y., O’donnell B.F. (2016). Resting-state EEG, impulsiveness, and personality in daily and nondaily smokers. Clin. Neurophysiol..

[29] Crüts B., van Etten L., Törnqvist H., Blomberg A., Sandström T., Mills N.L., Borm P.J. (2008). Exposure to diesel exhaust induces changes in EEG in human volunteers. Part. Fibre Toxicol..

[30] Gawryluk J.R., Palombo D.J., Curran J., Parker A., Carlsten C. (2023). Brief diesel exhaust exposure acutely impairs functional brain connectivity in humans: A randomized controlled crossover study. Environ. Health.

[31] Arnaldi D., Donniaquio A., Mattioli P., Massa F., Grazzini M., Meli R., Filippi L., Grisanti S., Famà F., Terzaghi M. (2020). Epilepsy in neurodegenerative dementias: A clinical, epidemiological, and EEG study. J. Alzheimer’s Dis..

[32] Bhat S., Acharya U.R., Dadmehr N., Adeli H. (2015). Clinical neurophysiological and automated EEG-based diagnosis of the Alzheimer’s disease. Eur. Neurol..

[33] Torkmahalleh M.A., Naseri M., Nurzhan S., Gabdrashova R., Bekezhankyzy Z., Gimnkhan A., Malekipirbazari M., Jouzizadeh M., Tabesh M., Farrokhi H. (2022). Human exposure to aerosol from indoor gas stove cooking and the resulting nervous system responses. Indoor Air.

[34] Naseri M., Jouzizadeh M., Tabesh M., Malekipirbazari M., Gabdrashova R., Nurzhan S., Farrokhi H., Khanbabaie R., Mehri-Dehnavi H., Bekezhankyzy Z. (2019). The impact of frying aerosol on human brain activity. Neurotoxicology.

[35] Longo V.D., Mattson M.P. (2014). Fasting: Molecular mechanisms and clinical applications. Cell Metab..

[36] Mattson M.P. (2012). Energy intake and exercise as determinants of brain health and vulnerability to injury and disease. Cell Me-Tabolism.

[37] Ooi T.C., Meramat A., Rajab N.F., Shahar S., Ismail I.S., Azam A.A., Sharif R. (2020). Intermittent fasting enhanced the cognitive function in older adults with mild cognitive impairment by in-ducing biochemical and metabolic changes: A 3-year progressive study. Nutrients.

[38] Currenti W., Godos J., Castellano S., Caruso G., Ferri R., Caraci F., Grosso G., Galvano F. (2021). Association between time restricted feeding and cognitive status in older Italian adults. Nutrients.

[39] Currenti W., Godos J., Castellano S., Caruso G., Ferri R., Caraci F., Grosso G., Galvano F. (2021). Time-restricted feeding is associated with mental health in elderly Italian adults. Chronobiol. Internatl..

[40] Höhn S., Dozières-Puyravel B., Auvin S. (2019). History of dietary treatment: Guelpa & Marie first report of intermittent fasting for epilepsy in 1911. Epilepsy Behav..

[41] Brocchi A., Rebelos E., Dardano A., Mantuano M., Daniele G. (2022). Effects of intermittent fasting on brain metabolism. Nutrients.

[42] Landgrave-Gómez J., Mercado-Gómez O.F., Vázquez-García M., Rodríguez-Molina V., Córdova-Dávalos L., Arriaga-Ávila V., Miranda-Martínez A., Guevara-Guzmán R. (2016). Anticonvulsant effect of time-restricted feeding in a pilocarpine-induced seizure model: Metabolic and epigenetic implications. Front. Cell Neurosci..

[43] Friedman M. (1937). The use of ranks to avoid the assumption of normality implicit in the analysis of variance. J. Am. Stat. Assoc..

[44] Wilcoxon F. (1945). Individual comparisons by ranking methods. Biom Bull.

[45] Torkmahalleh M.A., Ospanova S., Baibatyrova A., Nurbay S., Zhanakhmet G., Shah D. (2018). Contributions of burner, pan, meat and salt to PM emission during grilling. Environ. Res..

[46] Wallace L.A., Ott W.R., Weschler C.J. (2015). Ultrafine particles from electric appliances and cooking pans: Experiments suggesting desorp-tion/nucleation of sorbed organics as the primary source. Indoor Air.

[47] Buonanno G., Johnson G., Morawska L., Stabile L. (2011). Volatility Characterization of Cooking-Generated Aerosol Particles. Aerosol Sci. Technol..

[48] Cacot P., Tesolin B., Sebban C. (1995). Diurnal variations of EEG power in healthy adults. Electroencephalogr. Clin. Neurophysiol..

[49] Kaiser D.A. (2008). Ultradian and circadian effects in electroencephalography activity. Biofeedback.

[50] Preti M.G., Bolton T.A., Van De Ville D. (2017). The dynamic functional connectome: State-of-the-art and perspectives. Neuroimage.

[51] Cummings L., Dane A., Rhodes J., Lynch P., Hughes A.M. (2000). Diurnal variation in the quantitative EEG in healthy adult volunteers. Br. J. Clin. Pharmacol..

[52] Jeong J. (2004). EEG dynamics in patients with Alzheimer’s disease. Clin. Neurophysiol..

[53] Malek N., Baker M.R., Mann C., Greene J. (2017). Electroencephalographic markers in dementia. Acta Neurol. Scand..

[54] Micanovic C., Pal S. (2014). The diagnostic utility of EEG in early-onset dementia: A systematic review of the literature with narrative analysis. J. Neural Transm..

[55] Mientus S., Gallinat J., Wuebben Y., Pascual-Marqui R.D., Mulert C., Frick K., Dorn H., Herrmann W.M., Winterer G. (2002). Cortical hypoactivation during resting EEG in schizophrenics but not in depressives and schizotypal subjects as revealed by low resolution electromagnetic tomography (LORETA). Psychiatry Res. Neuroimaging.

[56] Rosén I. (1997). Electroencephalography as a diagnostic tool in dementia. Dement. Geriatr. Cogn. Disord..

[57] Soikkeli R., Partanen J., Soininen H., Pääkkönen A., Riekkinen P. (1991). Slowing of EEG in Parkinson’s disease. Electroencephalogr. Clin. Neurophysiol..

[58] Almeneessier A.S., BaHammam A.A., Olaish A.H., Pandi-Perumal S.R., Manzar D., BaHammam A.S. (2019). Effects of diurnal intermittent fasting on daytime sleepiness reflected by EEG absolute power. J. Clin. Neurophysiol..

[59] Xing Y.F., Xu Y.H., Shi M.H., Lian Y.X. (2016). The impact of PM2.5 on the human respiratory system. J. Thorac. Dis..

[60] Calderón-Garcidueñas L., Azzarelli B., Acuna H., Garcia R., Gambling T.M., Osnaya N., Monroy S., Tizapantzi M.D.R., Carson J.L., Villarreal-Calderon A. (2002). Air pollution and brain damage. Toxicol. Pathol..

[61] Elder A., Gelein R., Silva V., Feikert T., Opanashuk L., Carter J., Potter R., Maynard A., Ito Y., Finkelstein J. (2006). Translocation of inhaled ultrafine manganese oxide particles to the central nervous system. Environ. Health Perspect..

[62] Lin D.M., Ngai J. (1999). Development of the vertebrate main olfactory system. Curr. Opin. Neurobiol..

[63] Calderón-Garcidueñas L., Franco-Lira M., Torres-Jardón R., Henriquez-Roldán C., Barragán-Mejía G., Valencia-Salazar G., Gonzaléz-Maciel A., Reynoso-Robles R., Villarreal-Calderón R., Reed W. (2007). Pediatric respiratory and systemic effects of chronic air pollution exposure: Nose, lung, heart, and brain pathology. Toxicol. Pathol..

[64] Sohal R.S., Ku H.-H., Agarwal S., Forster M.J., Lal H. (1994). Oxidative damage, mitochondrial oxidant generation and antioxidant defenses during aging and in response to food restriction in the mouse. Mech. Ageing Dev..

[65] Wachsman J.T. (1996). The beneficial effects of dietary restriction: Reduced oxidative damage and enhanced apoptosis. Mutat. Res./Fundam. Mol. Mech. Mutagen..

[66] Sohal R.S., Weindruch R. (1996). Oxidative stress, caloric restriction, and aging. Sci. Total Environ..

[67] Fusco S., Pani G. (2013). Brain response to calorie restriction. Cell Mol. Life Sci..

[68] Kaptan Z., Akgün-Dar K., Kapucu A., Dedeakayoğulları H., Batu., Üzüm G. (2015). Long term consequences on spatial learning-memory of low-calorie diet during adolescence in female rats; hippocampal and prefrontal cortex BDNF level, expression of NeuN and cell proliferation in dentate gyrus. Brain Res..

[69] Marosi K., Mattson M.P. (2014). BDNF mediates adaptive brain and body responses to energetic challenges. Trends Endocrinol..

[70] Duan W., Guo Z., Jiang H., Ware M., Mattson M.P. (2003). Reversal of behavioral and metabolic abnormalities, and insulin resistance syndrome, by dietary restriction in mice deficient in brain-derived neurotrophic factor. Endocrinology.

[71] Lee J., Duan W., Mattson M.P. (2002). Evidence that brain-derived neurotrophic factor is required for basal neurogenesis and mediates, in part, the enhancement of neurogenesis by dietary restriction in the hippocampus of adult mice. J. Neurochem..

[72] Stranahan A.M., Lee K., Martin B., Maudsley S., Golden E., Cutler R.G., Mattson M.P. (2009). Voluntary exercise and caloric restriction enhance hippocampal dendritic spine density and BDNF levels in diabetic mice. Hippocampus.

[73] Johnson D.A., Johnson J.A. (2015). Nrf2—A therapeutic target for the treatment of neurodegenerative diseases. Free. Radic. Biol. Med..

[74] Singh R., Lakhanpal D., Kumar S., Sharma S., Kataria H., Kaur M., Kaur G. (2012). Late-onset intermittent fasting dietary restriction as a potential intervention to retard age-associated brain function impairments in male rats. Age.

[75] Cerqueira F.M., Cunha F.M., Laurindo F.R., Kowaltowski A.J. (2012). Calorie restriction increases cerebral mitochondrial respiratory capacity in a NO^•^-mediated mecha-nism: Impact on neuronal survival. Free Radic. Biol. Med..

