# Supplementary material for: Interaction of Cooking-Generated Aerosols on the Human Nervous System and the Impact of Caloric Restriction Post-Exposure
Source: Nutrients. 2024 Oct 17;16(20):3525. doi: 10.3390/nu16203525 (PMC11510529; doi:10.3390/nu16203525)
Supplement: Supplementary file 1 [file nutrients-16-03525-s001.zip › nutrients-3234976-supplementary.pdf]

*Supplementary Materials*

# Interaction of Cooking-Generated Aerosols on the Human Nervous System and the Impact of Caloric Restriction Post-Exposure

**Motahareh Naseri [1], Sahar Sadeghi [1], Milad Malekipirbazari [2], Sholpan Nurzhan [3], Raikhangul Gabdrashova [3], Zhibek Bekezhankyzy [4], Reza Khanbabaie [5], Byron Crape [6], Dhawal Shah [1,*] and Mehdi Amouei Torkmahalleh [7,*]**

1 Department of Chemical and Materials Engineering, School of Engineering and Digital Sciences, Nazarbayev University, Astana 010000, Kazakhstan; motahareh.naseri@nu.edu.kz (M.N.); sadeghi4527@gmail.com (S.S.)
2 Department of Computer Science and Engineering, Chalmers University of Technology and University of Gothenburg, SE-41296 Gothenburg, Sweden; milad.maleki@gmail.com
3 Department of Biological Sciences, School of Science and Humanities, Nazarbayev University, Astana 010000, Kazakhstan; sholpan.nurzhan@nu.edu.kz (S.N.); raikhangul.gabdrashova@nu.edu.kz (R.G.)
4 Department of Chemistry, School of Engineering, Nazarbayev University, Astana 010000, Kazakhstan; zhibek.bekezhankyzy@alumni.nu.edu.kz
5 Department of Physics, IKK Barber School of Arts and Sciences, University of British Columbia, Kelowna, BC V1V 1V7, Canada; reza.khanbabaie@gmail.com
6 Department of Biomedical Sciences, School of Medicine, Nazarbayev University, Astana 010000, Kazakhstan; byron.crape@nu.edu.kz
7 Division of Environmental and Occupational Health Sciences, School of Public Health, University of Illinois at Chicago, Chicago, IL 60612, USA
* Correspondence: dhawal.shah@nu.edu.kz (D.S.); mehdiat@uic.edu (M.A.T.)

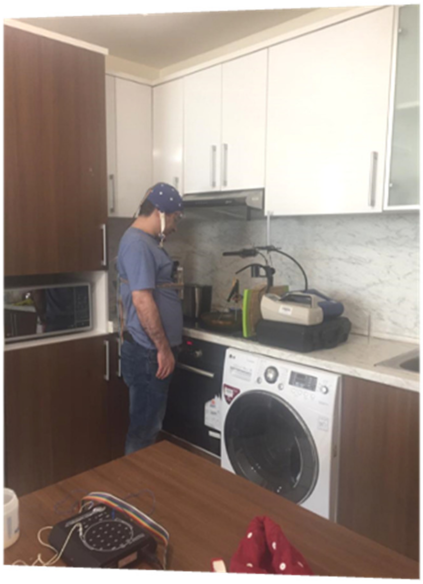

**Figure S1.** The test room setup.

Figure S2 illustrates the average of $CO_2$ concentrations throughout the cooking using an electric stove. Initially, $CO_2$ levels were around 900 ppm but gradually increased, hitting 980 ppm when the stove was switched off. $CO_2$ concentrations decreased after the stove was turned off, and reached background level.The data suggests that flipping or adding meat had minimal effect on $CO_2$ levels. Aside from the cooking itself, the presence of people in the room likely played a role in the $CO_2$ buildup observed during the experiment.

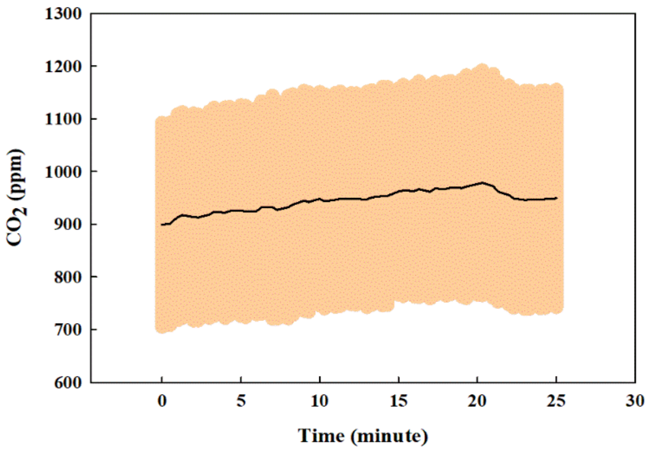


**Figure S2.** $CO_2$ concentration during cooking time (The black line represents the average values, while the yellow area indicates the standard deviation).

Figure S3 shows how indoor air temperature and relative humidity (RH) changed over time at the breathing zone above the pan. At the start, the room temperature was around 28°C. The temperature went through three phases: it rose sharply during the first 8 minutes before the kebabs were added, continued to increase gradually during cooking until minute 20 when the stove was turned off, and then remained steady with a slight decline toward the experiment's end. Initially, RH was about 32%. Over the first 8 minutes, it dropped to 28%, likely due to the rising air temperature. After the kebabs were added at minute 8, RH increased slightly, likely from the moisture released by the meat as temperatures climbed.

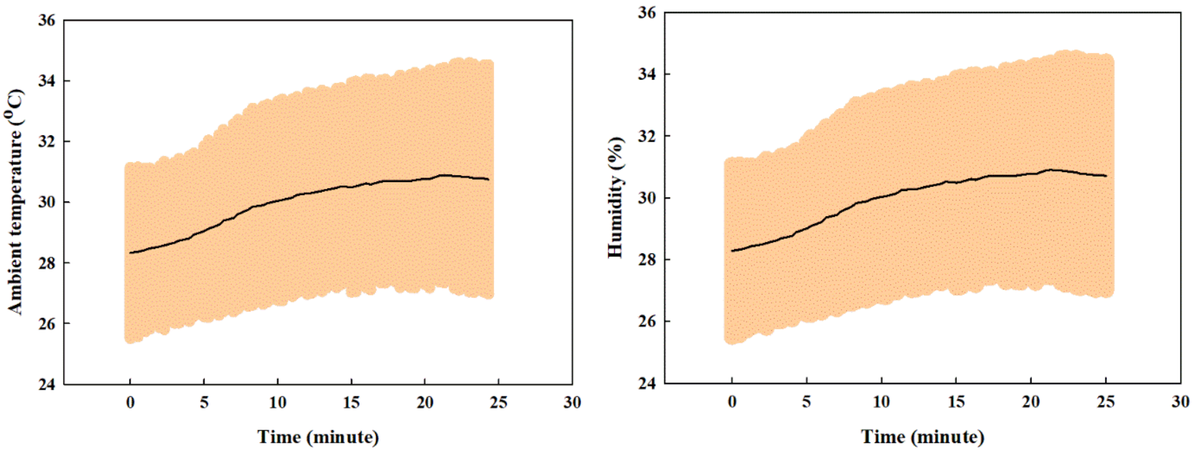


**Figure S3.** Room temperature and relative hmidity during cooking time (The black line represents the average values, while the yellow area indicates the standard deviation).

For the 2h-zero calorie group, there was an increasing trend for the alpha band and a decreasing trend for beta1, beta2, and beta3 in the whole brain; however, none were statistically significant. The increasing trend for relative power in delta band significantly changed in temporal ($RP_{step1}$ = 24.23 ± 8.08 and $RP_{step2}$ = 27.19 ± 10.77 with $RP_{step3}$ = 20.11 ± 5.49 and $RP_{step3}$ = 20.11 ± 5.49 with $RP_{step4}$ = 22.78 ± 6.84) and decreased significantly in the whole brain ($RP_{step1}$ = 26.13 ± 8.72 and $RP_{step2}$ = 27.64 ± 9.26 with $RP_{step3}$ = 22.26 ± 7.02). Theta band showed statistically significant decreased in left temporal ($RP_{step2}$ = 15.87 ± 2.82 with $RP_{step3}$ = 13.74 ± 4.67) and significant changes in temporal ($RP_{step2}$ = 15.44 ± 2.68 with $RP_{step3}$ = 12.76 ± 3.85 and $RP_{step3}$ = 12.76 ± 3.85 with $RP_{step4}$ = 14.16 ± 4.57). The observed changes in EEG frequency bands among the zero calorie group would be due to diurnal effects, zero calorie, and exposure to the cooking aerosol. Further analyses are needed to understand the impact of each factor on brain EEG.

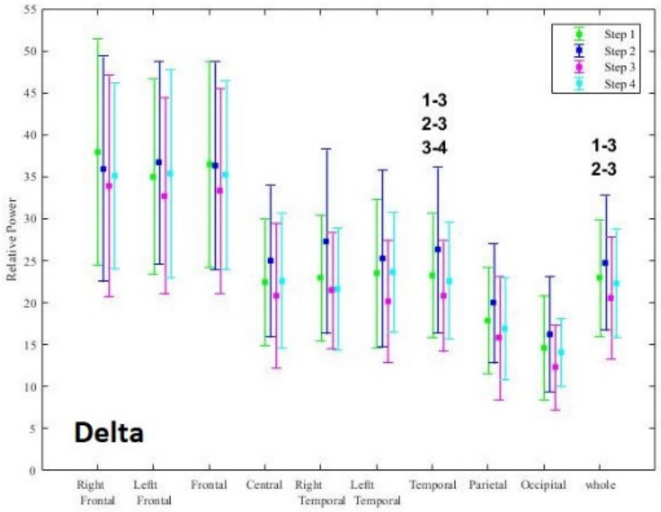


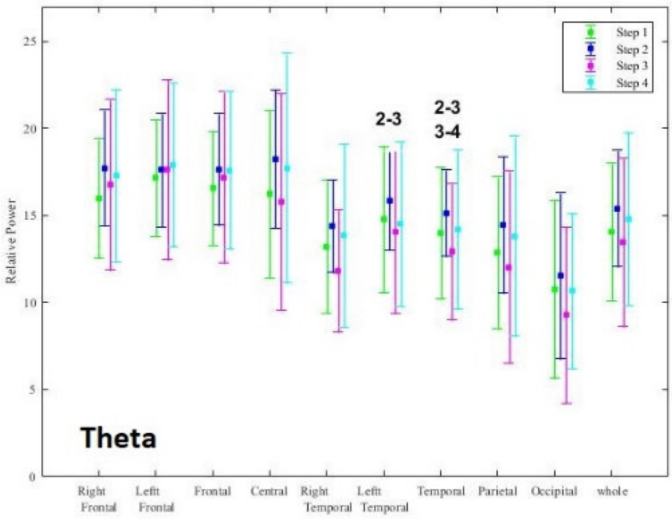


**Figure S4.** The variations of the delta and theta bands for different exposure periods for different lobes of the brain (2 h-zero calorie group) (The numbers on the bar graph indicate the stages that there was a statistically significant difference in relative power).

**Table S1.** The *p* values for all bands at all lobes during the control experiments for four different steps, including before cooking (step 1), 60 minutes after the cooking (step 2), 90 minutes after the cooking (step 3), and 120 minutes after the cooking (step 4).

| Band | Label | Friedman *p* Value | wilcox12. *p* Value | wilcox13. *p* Value | wilcox14 *p* Value | wilcox23 *p* Value | wilcox24 *p* Value | wilcox34 *p* Value |
|---|---|---|---|---|---|---|---|---|
| **delta** | Left.Frontal | 0.717296 | 0.382813 | 0.546875 | 0.195313 | 0.546875 | 0.945313 | 0.460938 |
| **delta** | Right.Frontal | 0.440227 | 0.546875 | 0.84375 | 0.054688 | 0.546875 | 0.460938 | 0.148438 |
| **delta** | Frontal | 0.68227 | 0.382813 | 0.84375 | 0.054688 | 0.84375 | 0.742188 | 0.25 |
| **delta** | Left.Temporal | 0.164619 | 0.640625 | 0.460938 | 0.078125 | 0.25 | 0.054688 | 0.460938 |
| **delta** | Right.Temporal | 0.68227 | 0.546875 | 0.640625 | 0.640625 | 0.195313 | 0.078125 | 0.640625 |
| **delta** | Temporal | 0.391625 | 0.460938 | 0.546875 | 0.25 | 0.148438 | 0.054688 | 0.546875 |
| **delta** | Central | 0.896432 | 0.460938 | 0.640625 | 0.546875 | 1.0 | 0.3125 | 0.3125 |
| **delta** | Parietal | 0.289756 | 0.148438 | 0.742188 | 0.742188 | 0.3125 | 0.109375 | 0.640625 |
| **delta** | Occipital | 0.466323 | 0.148438 | 0.546875 | 0.640625 | 0.3125 | 0.148438 | 0.84375 |
| **delta** | Whole.Band | 0.717296 | 0.640625 | 0.945313 | 0.25 | 0.640625 | 0.195313 | 0.382813 |
| **theta** | Left.Frontal | 0.960028 | 0.84375 | 0.3125 | 0.382813 | 0.84375 | 0.546875 | 0.382813 |
| **theta** | Right.Frontal | 0.861385 | 0.640625 | 0.460938 | 0.742188 | 0.84375 | 0.25 | 0.640625 |
| **theta** | Frontal | 0.717296 | 0.640625 | 0.382813 | 0.546875 | 0.945313 | 0.382813 | 0.3125 |

| | | | | | | | | |
|---|---|---|---|---|---|---|---|---|
| **theta** | Left.Temporal | 0.717296 | 0.84375 | 0.742188 | 0.25 | 0.460938 | 0.84375 | 0.742188 |
| **theta** | Right.Temporal | 0.789156 | 0.640625 | 0.546875 | 0.742188 | 0.546875 | 0.148438 | 0.078125 |
| **theta** | Temporal | 0.960028 | 0.84375 | 0.742188 | 0.460938 | 0.742188 | 0.460938 | 0.546875 |
| **theta** | Central | 0.522167 | 0.3125 | 0.382813 | 0.84375 | 0.742188 | 0.078125 | 0.640625 |
| **theta** | Parietal | 0.717296 | 0.3125 | 0.382813 | 0.382813 | 0.546875 | 0.195313 | 0.546875 |
| **theta** | Occipital | 0.753004 | 0.460938 | 0.460938 | 0.25 | 0.460938 | 0.742188 | 0.742188 |
| **theta** | Whole.Band | 0.929731 | 0.3125 | 0.546875 | 0.640625 | 0.945313 | 0.460938 | 0.460938 |
| **alpha** | Left.Frontal | 0.582849 | 0.945313 | 1.0 | 0.640625 | 0.945313 | 0.84375 | 0.546875 |
| **alpha** | Right.Frontal | 0.648107 | 0.640625 | 0.84375 | 0.382813 | 0.84375 | 0.460938 | 0.25 |
| **alpha** | Frontal | 0.648107 | 0.84375 | 1.0 | 0.546875 | 0.84375 | 0.546875 | 0.25 |
| **alpha** | Left.Temporal | 0.522167 | 0.460938 | 0.742188 | 0.742188 | 0.460938 | 0.25 | 0.382813 |
| **alpha** | Right.Temporal | 0.369071 | 0.25 | 0.84375 | 1.0 | 0.195313 | 0.148438 | 0.84375 |
| **alpha** | Temporal | 0.440227 | 0.382813 | 0.945313 | 0.742188 | 0.25 | 0.148438 | 0.640625 |
| **alpha** | Central | 0.415335 | 0.382813 | 0.640625 | 0.742188 | 0.945313 | 0.195313 | 0.148438 |
| **alpha** | Parietal | 0.369071 | 0.195313 | 0.640625 | 0.25 | 0.382813 | 0.25 | 0.3125 |
| **alpha** | Occipital | 0.648107 | 0.195313 | 0.460938 | 0.3125 | 0.382813 | 0.460938 | 0.382813 |
| **alpha** | Whole.Band | 0.522167 | 0.382813 | 0.742188 | 0.945313 | 0.640625 | 0.25 | 0.25 |
| **beta1** | Left.Frontal | 0.091655 | 0.078125 | 0.054688 | 0.015625 | 0.84375 | 0.382813 | 0.382813 |
| **beta1** | Right.Frontal | 0.080308 | 0.25 | 0.078125 | 0.015625 | 1.0 | 0.25 | 0.3125 |
| **beta1** | Frontal | 0.050331 | 0.109375 | 0.054688 | 0.015625 | 1.0 | 0.195313 | 0.3125 |
| **beta1** | Left.Temporal | 0.006423 | 0.078125 | 0.007813 | 0.039063 | 0.023438 | 0.148438 | 0.078125 |
| **beta1** | Right.Temporal | 0.256115 | 0.148438 | 0.109375 | 0.039063 | 0.945313 | 0.546875 | 0.546875 |
| **beta1** | Temporal | 0.01694 | 0.109375 | 0.015625 | 0.039063 | 0.054688 | 0.25 | 0.148438 |
| **beta1** | Central | 0.144744 | 0.742188 | 0.3125 | 0.3125 | 0.054688 | 0.015625 | 1.0 |
| **beta1** | Parietal | 0.050331 | 0.742188 | 0.078125 | 0.109375 | 0.023438 | 0.023438 | 0.460938 |
| **beta1** | Occipital | 0.256115 | 0.460938 | 0.054688 | 0.460938 | 0.195313 | 0.742188 | 0.054688 |
| **beta1** | Whole.Band | 0.050331 | 0.382813 | 0.023438 | 0.023438 | 0.078125 | 0.109375 | 0.460938 |
| **beta2** | Left.Frontal | 0.091655 | 0.039063 | 0.023438 | 0.078125 | 0.84375 | 1.0 | 0.546875 |
| **beta2** | Right.Frontal | 0.080308 | 0.109375 | 0.109375 | 0.015625 | 0.640625 | 0.3125 | 0.742188 |
| **beta2** | Parietal | 0.327305 | 0.742188 | 0.195313 | 0.109375 | 0.546875 | 0.84375 | 0.546875 |
| **beta2** | Occipital | 0.960028 | 0.84375 | 0.25 | 0.460938 | 1.0 | 0.546875 | 0.945313 |
| **beta2** | Whole.Band | 0.080308 | 0.3125 | 0.007813 | 0.039063 | 0.460938 | 0.460938 | 0.742188 |
| **beta3** | Left.Frontal | 0.11161 | 0.078125 | 0.023438 | 0.382813 | 0.742188 | 0.742188 | 0.945313 |
| **beta3** | Right.Frontal | 0.240662 | 0.078125 | 0.109375 | 0.078125 | 0.25 | 0.460938 | 0.382813 |
| **beta3** | Frontal | 0.080308 | 0.078125 | 0.023438 | 0.054688 | 0.546875 | 0.640625 | 0.84375 |
| **beta3** | Left.Temporal | 0.415335 | 0.742188 | 0.84375 | 0.3125 | 0.546875 | 0.25 | 0.742188 |
| **beta3** | Right.Temporal | 0.369071 | 0.546875 | 0.742188 | 0.148438 | 0.945313 | 0.460938 | 0.3125 |
| **beta3** | Temporal | 0.391625 | 0.640625 | 0.945313 | 0.25 | 0.742188 | 0.25 | 0.382813 |
| **beta3** | Central | 0.985226 | 0.742188 | 0.546875 | 0.945313 | 0.945313 | 0.84375 | 0.640625 |
| **beta3** | Parietal | 0.929731 | 0.84375 | 0.546875 | 0.945313 | 0.460938 | 0.460938 | 0.640625 |
| **beta3** | Whole.Band | 0.256115 | 0.148438 | 0.195313 | 0.148438 | 0.945313 | 0.382813 | 0.640625 |

**Table S2.** The *p* values calculated from comparing the RP values across different post-exposure steps registered for all electrodes and frequency bands among the 2 h-zero calorie intake group.

| Attribute | Friedman *p* Value | wilcox12 *p* Value | wilcox13 *p* Value | wilcox14 *p* Value | wilcox23 *p* Value | wilcox24 *p* Value | wilcox34 *p* Value |
|---|---|---|---|---|---|---|---|
| **Fp1_delta** | 0.794339 | 0.951538 | 0.760864 | 0.541626 | 0.625732 | 0.463135 | 0.118896 |
| **Fp2_delta** | 0.652929 | 0.172607 | 0.42627 | 0.903198 | 0.807739 | 0.193726 | 0.325806 |
| **F3_delta** | 0.297462 | 0.267578 | 0.625732 | 0.807739 | 0.090576 | 0.295776 | 0.463135 |
| **F4_delta** | 0.201105 | 0.760864 | 0.118896 | 0.583008 | 0.020264 | 0.714844 | 0.325806 |
| **F7_delta** | 0.216145 | 0.760864 | 0.135254 | 0.216553 | 0.153076 | 0.267578 | 0.6698 |
| **F8_delta** | 0.318914 | 0.390991 | 0.241211 | 0.090576 | 0.153076 | 0.057983 | 0.6698 |
| **Fz_delta** | 0.258395 | 0.714844 | 0.104004 | 0.714844 | 0.153076 | 0.807739 | 0.153076 |
| **C3_delta** | 0.224049 | 0.463135 | 0.104004 | 0.951538 | 0.016602 | 0.760864 | 0.153076 |
| **C4_delta** | 0.18036 | 0.118896 | 0.153076 | 0.855225 | 0.008545 | 0.267578 | 0.078491 |
| **Cz_delta** | 0.365942 | 0.625732 | 0.118896 | 0.541626 | 0.049438 | 0.807739 | 0.090576 |
| **P3_delta** | 0.011421 | 0.541626 | 0.04187 | 1.0 | 0.001709 | 0.193726 | 0.006714 |
| **P4_delta** | 0.038429 | 0.714844 | 0.024536 | 0.42627 | 0.005249 | 0.078491 | 0.078491 |
| **Pz_delta** | 0.794339 | 0.541626 | 0.295776 | 0.903198 | 0.216553 | 0.267578 | 0.325806 |
| **O1_delta** | 0.092522 | 0.267578 | 0.013428 | 1.0 | 0.005249 | 0.193726 | 0.104004 |
| **O2_delta** | 0.039944 | 1.0 | 0.04187 | 0.501587 | 0.016602 | 1.0 | 0.035278 |
| **T3_delta** | 0.085801 | 0.583008 | 0.057983 | 1.0 | 0.004028 | 0.583008 | 0.153076 |
| **T4_delta** | 0.712245 | 0.714844 | 0.390991 | 1.0 | 0.029541 | 0.267578 | 0.583008 |
| **T5_delta** | 0.18036 | 0.855225 | 0.078491 | 0.760864 | 0.003052 | 0.855225 | 0.013428 |
| **T6_delta** | 0.01236 | 0.295776 | 0.216553 | 0.267578 | 0.008545 | 0.006714 | 0.903198 |
| **Fp1_theta** | 0.835756 | 0.501587 | 0.903198 | 0.903198 | 0.625732 | 0.760864 | 0.501587 |
| **Fp2_theta** | 0.124813 | 0.104004 | 0.325806 | 0.541626 | 0.295776 | 0.357544 | 0.807739 |
| **F3_theta** | 0.391625 | 0.135254 | 0.903198 | 0.625732 | 0.216553 | 0.501587 | 0.855225 |
| **F4_theta** | 0.477878 | 0.104004 | 0.855225 | 0.216553 | 0.42627 | 1.0 | 0.241211 |
| **F7_theta** | 0.773627 | 0.625732 | 0.501587 | 0.501587 | 0.714844 | 0.951538 | 0.760864 |
| **C3_theta** | 0.124813 | 0.049438 | 0.807739 | 0.42627 | 0.090576 | 0.625732 | 0.390991 |
| **C4_theta** | 0.144744 | 0.118896 | 0.903198 | 0.135254 | 0.04187 | 0.903198 | 0.013428 |
| **Cz_theta** | 0.155805 | 0.035278 | 0.541626 | 0.104004 | 0.049438 | 0.541626 | 0.135254 |
| **P3_theta** | 0.025063 | 0.118896 | 0.325806 | 0.42627 | 0.020264 | 0.267578 | 0.010742 |
| **P4_theta** | 0.054345 | 0.216553 | 0.625732 | 0.118896 | 0.035278 | 0.855225 | 0.006714 |
| **Pz_theta** | 0.018325 | 0.104004 | 0.760864 | 0.216553 | 0.029541 | 0.807739 | 0.067627 |
| **O1_theta** | 0.40503 | 0.760864 | 0.325806 | 0.855225 | 0.153076 | 0.357544 | 0.135254 |
| **O2_theta** | 0.099748 | 0.463135 | 0.193726 | 0.295776 | 0.067627 | 0.714844 | 0.024536 |
| **T3_theta** | 0.16766 | 0.067627 | 0.42627 | 0.760864 | 0.135254 | 0.267578 | 0.714844 |
| **T4_theta** | 0.092522 | 0.078491 | 0.193726 | 0.42627 | 0.020264 | 0.463135 | 0.020264 |
| **T5_theta** | 0.079551 | 0.541626 | 0.295776 | 1.0 | 0.078491 | 0.118896 | 0.172607 |
| **T6_theta** | 0.103561 | 0.463135 | 0.010742 | 0.501587 | 0.024536 | 0.42627 | 0.035278 |
| **Fp1_alpha** | 0.297462 | 0.714844 | 0.357544 | 0.42627 | 0.42627 | 0.463135 | 0.057983 |
| **Fp2_alpha** | 0.287227 | 0.541626 | 0.267578 | 0.951538 | 0.463135 | 0.501587 | 0.135254 |
| **F3_alpha** | 0.596462 | 0.357544 | 1.0 | 0.390991 | 0.541626 | 0.714844 | 0.463135 |
| **F4_alpha** | 0.365942 | 0.583008 | 0.357544 | 0.325806 | 0.172607 | 0.951538 | 0.153076 |
| **F7_alpha** | 0.155805 | 0.855225 | 0.104004 | 0.807739 | 0.172607 | 0.325806 | 0.325806 |
| **F8_alpha** | 0.249385 | 0.855225 | 0.241211 | 0.6698 | 0.216553 | 0.390991 | 0.501587 |
| **Fz_alpha** | 0.224049 | 0.807739 | 0.104004 | 0.807739 | 0.049438 | 0.463135 | 0.216553 |
| **C3_alpha** | 0.297462 | 0.357544 | 0.501587 | 0.42627 | 0.049438 | 0.463135 | 0.090576 |
| **C4_alpha** | 0.124813 | 0.193726 | 0.357544 | 0.104004 | 0.008545 | 0.807739 | 0.016602 |
| **Cz_alpha** | 0.543291 | 0.390991 | 0.541626 | 0.267578 | 0.049438 | 0.625732 | 0.078491 |
| **P3_alpha** | 0.036971 | 0.42627 | 0.118896 | 0.625732 | 0.013428 | 0.357544 | 0.005249 |

| | | | | | | | |
|---|---|---|---|---|---|---|---|
| **P4_alpha** | 0.11161 | 0.583008 | 0.135254 | 0.42627 | 0.024536 | 0.267578 | 0.013428 |
| **Pz_alpha** | 0.115856 | 0.390991 | 0.42627 | 0.267578 | 0.216553 | 0.807739 | 0.035278 |
| **O1_alpha** | 0.216145 | 0.583008 | 0.090576 | 1.0 | 0.020264 | 0.357544 | 0.024536 |
| **O2_alpha** | 0.107514 | 0.760864 | 0.067627 | 1.0 | 0.057983 | 0.583008 | 0.024536 |
| **T3_alpha** | 0.330145 | 0.903198 | 0.193726 | 0.903198 | 0.035278 | 1.0 | 0.153076 |
| **T4_alpha** | 0.240662 | 1.0 | 0.42627 | 0.903198 | 0.104004 | 0.501587 | 0.325806 |
| **T5_alpha** | 0.070993 | 0.951538 | 0.135254 | 1.0 | 0.010742 | 0.6698 | 0.020264 |
| **T6_alpha** | 0.032913 | 0.325806 | 0.067627 | 0.625732 | 0.008545 | 0.049438 | 0.104004 |
| **Fp1beta1** | 0.596462 | 0.357544 | 0.714844 | 0.325806 | 0.067627 | 0.583008 | 0.501587 |
| **Fp2beta1** | 0.652929 | 0.625732 | 0.807739 | 0.463135 | 0.6698 | 0.135254 | 0.135254 |
| **F3_beta1** | 0.44756 | 0.216553 | 0.501587 | 1.0 | 0.903198 | 0.501587 | 0.172607 |
| **F4_beta1** | 0.732528 | 0.153076 | 0.855225 | 0.760864 | 0.325806 | 0.541626 | 0.295776 |
| **F7_beta1** | 0.896432 | 0.42627 | 0.541626 | 0.855225 | 0.714844 | 0.6698 | 0.903198 |
| **F8_beta1** | 0.692196 | 0.714844 | 0.541626 | 0.855225 | 0.135254 | 0.267578 | 1.0 |
| **Fz_beta1** | 0.818652 | 0.583008 | 0.216553 | 0.357544 | 0.944285 | 0.807739 | 0.541626 |
| **C3_beta1** | 0.934279 | 0.42627 | 0.390991 | 0.6698 | 0.951538 | 0.903198 | 0.903198 |
| **C4_beta1** | 0.44756 | 0.42627 | 0.583008 | 0.325806 | 0.463135 | 0.241211 | 0.216553 |
| **Cz_beta1** | 0.856288 | 0.216553 | 0.42627 | 0.6698 | 0.6698 | 0.357544 | 0.760864 |
| **P3_beta1** | 0.50979 | 0.078491 | 0.172607 | 0.6698 | 0.903198 | 0.295776 | 0.172607 |
| **P4_beta1** | 0.773627 | 0.541626 | 0.325806 | 0.855225 | 0.625732 | 0.951538 | 0.463135 |
| **Pz_beta1** | 0.070993 | 0.172607 | 0.04187 | 0.807739 | 0.295776 | 0.390991 | 0.010742 |
| **O1_beta1** | 0.44756 | 0.172607 | 0.325806 | 0.6698 | 0.501587 | 0.325806 | 0.029541 |
| **O2_beta1** | 0.232219 | 0.172607 | 0.104004 | 0.501587 | 0.583008 | 0.903198 | 0.325806 |
| **T3_beta1** | 0.896432 | 0.583008 | 0.807739 | 0.951538 | 0.760864 | 0.807739 | 0.501587 |
| **T4_beta1** | 0.16766 | 0.090576 | 0.153076 | 0.267578 | 0.267578 | 0.625732 | 0.583008 |
| **T5_beta1** | 0.815075 | 0.135254 | 0.501587 | 0.463135 | 1.0 | 0.714844 | 0.541626 |
| **T6_beta1** | 0.773627 | 0.135254 | 0.760864 | 0.390991 | 1.0 | 0.903198 | 0.760864 |
| **Fp1beta2** | 0.815075 | 0.714844 | 0.855225 | 0.583008 | 0.903198 | 0.951538 | 0.583008 |
| **Fp2_beta** | 0.40503 | 0.153076 | 1.0 | 0.583008 | 0.325806 | 0.104004 | 0.541626 |
| **F3_beta2** | 0.216145 | 0.295776 | 0.325806 | 0.104004 | 0.135254 | 0.118896 | 0.903198 |
| **F4_beta2** | 0.526343 | 0.325806 | 0.193726 | 0.118896 | 0.104004 | 0.267578 | 1.0 |
| **F7_beta2** | 0.896432 | 0.714844 | 0.714844 | 0.625732 | 0.760864 | 0.951538 | 0.807739 |
| **F8_beta2** | 0.365942 | 0.463135 | 0.903198 | 0.760864 | 0.078491 | 0.193726 | 0.172607 |
| **Fz_beta2** | 0.432993 | 0.807739 | 0.583008 | 0.357544 | 0.463135 | 0.501587 | 1.0 |
| **C3_beta2** | 0.692196 | 0.501587 | 1.0 | 0.6698 | 0.807739 | 0.583008 | 0.078491 |
| **C4_beta2** | 0.856288 | 0.541626 | 0.714844 | 0.625732 | 0.807739 | 0.193726 | 0.714844 |
| **Cz_beta2** | 0.993495 | 0.541626 | 1.0 | 0.714844 | 1.0 | 0.501587 | 0.42627 |
| **P3_beta2** | 0.070993 | 0.241211 | 0.067627 | 0.855225 | 0.903198 | 0.090576 | 0.067627 |
| **P4_beta2** | 0.815075 | 0.267578 | 0.463135 | 0.295776 | 0.714844 | 0.951538 | 0.583008 |
| **Pz_beta2** | 0.652929 | 0.951538 | 0.855225 | 1.0 | 0.903198 | 0.903198 | 0.118896 |
| **O1_beta2** | 0.063328 | 0.057983 | 0.024536 | 0.6698 | 0.855225 | 0.267578 | 0.020264 |
| **O2_beta2** | 0.155805 | 0.016602 | 0.153076 | 0.172607 | 0.583008 | 1.0 | 0.625732 |
| **T3_beta2** | 0.993495 | 0.807739 | 1.0 | 0.6698 | 0.541626 | 0.583008 | 0.760864 |
| **T4_beta2** | 0.240662 | 0.049438 | 0.714844 | 0.714844 | 0.193726 | 0.035278 | 0.903198 |
| **T5_beta2** | 0.208499 | 0.118896 | 0.193726 | 0.541626 | 0.390991 | 0.172607 | 0.390991 |
| **T6_beta2** | 0.099748 | 0.172607 | 0.153076 | 0.118896 | 0.42627 | 1.0 | 0.241211 |
| **Fp1beta3** | 0.50979 | 0.807739 | 0.463135 | 0.501587 | 1.0 | 0.951538 | 0.760864 |
| **Fp2beta3** | 0.477878 | 0.903198 | 0.135254 | 0.135254 | 0.42627 | 0.714844 | 0.855225 |
| **F3_beta3** | 0.418818 | 0.172607 | 0.625732 | 0.42627 | 0.855225 | 0.501587 | 0.325806 |
| **F4_beta3** | 0.856288 | 0.153076 | 0.625732 | 0.951538 | 0.463135 | 0.325806 | 0.153076 |
| **F7_beta3** | 0.652929 | 0.135254 | 0.807739 | 0.760864 | 0.714844 | 0.463135 | 0.903198 |

| | | | | | | | |
|---|---|---|---|---|---|---|---|
| **F8_beta3** | 0.493635 | 0.807739 | 0.807739 | 0.090576 | 0.42627 | 0.049438 | 0.295776 |
| **Fz_beta3** | 0.418818 | 1.0 | 0.153076 | 0.193726 | 0.090576 | 0.357544 | 0.42627 |
| **C3_beta3** | 0.692196 | 0.216553 | 0.42627 | 0.241211 | 1.0 | 0.625732 | 0.903198 |
| **C4_beta3** | 0.365942 | 0.216553 | 0.153076 | 0.6698 | 0.951538 | 0.903198 | 0.090576 |
| **Cz_beta3** | 0.18036 | 0.501587 | 0.067627 | 0.267578 | 0.267578 | 0.6698 | 0.001709 |
| **P3_beta3** | 0.493635 | 0.760864 | 0.172607 | 0.541626 | 0.583008 | 1.0 | 0.078491 |
| **P4_beta3** | 0.652929 | 0.760864 | 0.104004 | 0.625732 | 0.325806 | 0.501587 | 0.241211 |
| **Pz_beta3** | 0.578358 | 0.951538 | 0.295776 | 0.807739 | 0.390991 | 0.807739 | 0.104004 |
| **O1_beta3** | 0.277309 | 0.357544 | 0.035278 | 0.714844 | 0.267578 | 0.6698 | 0.010742 |
| **O2_beta3** | 0.013376 | 0.153076 | 0.001709 | 0.078491 | 0.078491 | 0.583008 | 0.067627 |
| **T3_beta3** | 0.614935 | 0.118896 | 0.714844 | 0.714844 | 0.267578 | 0.714844 | 0.807739 |
| **T4_beta3** | 0.173901 | 0.049438 | 0.760864 | 0.295776 | 0.118896 | 0.390991 | 0.760864 |
| **T5_beta3** | 0.44756 | 0.118896 | 0.104004 | 0.6698 | 0.855225 | 0.714844 | 0.295776 |
| **T6_beta3** | 0.085801 | 0.583008 | 0.005249 | 0.135254 | 0.357544 | 0.501587 | 0.172607 |

**Table S3.** The p values calculated from comparing the RP values across different post-exposure steps registered for all electrodes and frequency bands among the non-zero calorie intake group.

| Attribute | Friedman *p* Value | wilcox12 *p* Value | wilcox13 *p* Value | wilcox14 *p* Value | wilcox23 *p* Value | wilcox24 *p* Value | wilcox34 *p* Value |
|---|---|---|---|---|---|---|---|
| **Fp1_delta** | 0.650354 | 0.302795 | 0.488708 | 0.421204 | 0.803955 | 0.678772 | 0.719727 |
| **Fp2_delta** | 0.13984 | 0.524475 | 0.761536 | 0.187622 | 0.421204 | 0.599487 | 0.106995 |
| **F3_delta** | 0.614935 | 0.454285 | 0.389404 | 0.488708 | 0.454285 | 0.389404 | 0.072998 |
| **F4_delta** | 0.801252 | 0.330261 | 0.488708 | 0.488708 | 0.187622 | 0.389404 | 0.359131 |
| **F7_delta** | 0.356081 | 0.25238 | 0.151428 | 0.719727 | 0.846924 | 0.638672 | 0.276855 |
| **F8_delta** | 0.092059 | 0.454285 | 0.120544 | 0.135376 | 0.302795 | 0.083252 | 0.002014 |
| **Fz_delta** | 0.356081 | 0.934082 | 0.120544 | 0.761536 | 0.106995 | 0.302795 | 0.25238 |
| **C3_delta** | 0.13984 | 0.846924 | 0.012451 | 0.25238 | 0.072998 | 0.106995 | 0.120544 |
| **C4_delta** | 0.486232 | 0.421204 | 0.488708 | 0.803955 | 0.083252 | 0.168823 | 0.846924 |
| **Cz_delta** | 0.171797 | 0.168823 | 0.25238 | 0.389404 | 0.094604 | 0.030151 | 1.0 |
| **P3_delta** | 0.240662 | 0.846924 | 0.389404 | 0.151428 | 0.276855 | 0.063721 | 0.488708 |
| **P4_delta** | 0.303053 | 0.638672 | 0.561401 | 0.276855 | 0.276855 | 0.187622 | 0.599487 |
| **Pz_delta** | 0.196786 | 0.934082 | 0.389404 | 0.168823 | 0.187622 | 0.063721 | 0.488708 |
| **O1_delta** | 0.102275 | 0.761536 | 0.047913 | 0.035339 | 0.761536 | 0.168823 | 0.524475 |
| **O2_delta** | 0.092059 | 0.719727 | 0.055359 | 0.072998 | 0.135376 | 0.106995 | 0.454285 |
| **T3_delta** | 0.183895 | 0.678772 | 0.120544 | 1.0 | 0.035339 | 0.846924 | 0.207764 |
| **T4_delta** | 0.564146 | 0.719727 | 0.330261 | 0.934082 | 0.025574 | 0.421204 | 0.454285 |
| **T5_delta** | 0.232773 | 0.934082 | 0.25238 | 0.187622 | 0.187622 | 0.094604 | 0.846924 |
| **T6_delta** | 0.274717 | 0.599487 | 0.302795 | 0.055359 | 0.977966 | 0.168823 | 0.359131 |
| **Fp1_theta** | 0.650354 | 0.599487 | 0.330261 | 0.106995 | 0.846924 | 0.454285 | 0.890381 |
| **Fp2_theta** | 0.762613 | 0.934082 | 0.638672 | 0.207764 | 0.846924 | 0.302795 | 0.678772 |
| **F3_theta** | 0.257177 | 0.846924 | 0.207764 | 0.047913 | 0.803955 | 0.276855 | 0.454285 |
| **F4_theta** | 0.486232 | 0.524475 | 0.561401 | 0.063721 | 0.890381 | 0.421204 | 0.761536 |
| **F7_theta** | 0.404125 | 0.488708 | 0.638672 | 0.168823 | 0.330261 | 0.25238 | 0.488708 |
| **F8_theta** | 0.113573 | 0.359131 | 0.094604 | 0.021545 | 1.0 | 0.330261 | 0.359131 |
| **Fz_theta** | 0.257177 | 0.421204 | 0.25238 | 0.04126 | 0.977966 | 0.524475 | 0.803955 |
| **C3_theta** | 0.650354 | 0.599487 | 0.151428 | 0.803955 | 0.389404 | 0.488708 | 0.302795 |
| **C4_theta** | 0.257177 | 0.678772 | 0.094604 | 0.094604 | 0.302795 | 0.421204 | 0.803955 |
| **Cz_theta** | 0.724389 | 0.638672 | 0.25238 | 0.389404 | 0.977966 | 0.890381 | 0.678772 |
| **P3_theta** | 0.724389 | 0.977966 | 0.719727 | 0.359131 | 0.846924 | 0.389404 | 0.934082 |
| **P4_theta** | 0.155043 | 0.638672 | 0.106995 | 0.055359 | 0.638672 | 0.302795 | 0.977966 |
| **Pz_theta** | 0.948376 | 0.890381 | 0.302795 | 0.302795 | 0.761536 | 0.561401 | 0.890381 |

| | | | | | | | |
|---|---|---|---|---|---|---|---|
| **O1_theta** | 0.839878 | 0.638672 | 0.276855 | 0.168823 | 0.638672 | 0.276855 | 0.719727 |
| **O2_theta** | 0.257177 | 0.524475 | 0.207764 | 0.035339 | 0.330261 | 0.168823 | 0.678772 |
| **T3_theta** | 0.516363 | 0.803955 | 0.561401 | 0.977966 | 0.25238 | 0.719727 | 0.890381 |
| **T4_theta** | 0.064629 | 0.488708 | 0.015076 | 0.106995 | 0.229309 | 0.168823 | 0.524475 |
| **T5_theta** | 0.232773 | 0.25238 | 0.229309 | 0.055359 | 0.454285 | 0.934082 | 0.330261 |
| **T6_theta** | 0.092059 | 0.421204 | 0.010254 | 0.047913 | 0.803955 | 0.359131 | 0.803955 |
| **Fp1_alpha** | 0.762613 | 0.561401 | 0.488708 | 0.761536 | 0.330261 | 0.561401 | 0.934082 |
| **Fp2_alpha** | 0.547877 | 0.638672 | 0.977966 | 0.761536 | 0.421204 | 0.638672 | 0.934082 |
| **F3_alpha** | 0.486232 | 0.599487 | 0.276855 | 0.168823 | 0.761536 | 0.421204 | 0.934082 |
| **F4_alpha** | 0.160449 | 0.803955 | 0.302795 | 0.072998 | 0.761536 | 0.083252 | 0.421204 |
| **F7_alpha** | 0.121757 | 0.302795 | 0.047913 | 0.083252 | 0.330261 | 0.421204 | 0.846924 |
| **F8_alpha** | 0.547877 | 0.561401 | 0.302795 | 0.934082 | 0.229309 | 0.454285 | 0.389404 |
| **Fz_alpha** | 0.313063 | 0.803955 | 0.330261 | 0.135376 | 0.846924 | 0.187622 | 0.638672 |
| **C3_alpha** | 0.356081 | 0.638672 | 0.018066 | 0.168823 | 0.330261 | 0.359131 | 0.524475 |
| **C4_alpha** | 0.196786 | 0.678772 | 0.207764 | 0.135376 | 0.561401 | 0.106995 | 0.389404 |
| **Cz_alpha** | 0.232773 | 0.890381 | 0.719727 | 0.151428 | 0.561401 | 0.187622 | 0.454285 |
| **P3_alpha** | 0.274717 | 0.846924 | 0.761536 | 0.229309 | 0.846924 | 0.151428 | 0.389404 |
| **P4_alpha** | 0.356081 | 0.934082 | 0.561401 | 0.135376 | 0.561401 | 0.229309 | 0.389404 |
| **Pz_alpha** | 0.391625 | 0.761536 | 0.359131 | 0.229309 | 0.719727 | 0.302795 | 0.524475 |
| **O1_alpha** | 0.160449 | 0.977966 | 0.421204 | 0.04126 | 0.846924 | 0.168823 | 0.421204 |
| **O2_alpha** | 0.069391 | 1.0 | 0.207764 | 0.012451 | 0.302795 | 0.151428 | 0.488708 |
| **T3_alpha** | 0.042054 | 0.389404 | 0.025574 | 0.072998 | 0.454285 | 0.187622 | 0.846924 |
| **T4_alpha** | 0.257177 | 1.0 | 0.229309 | 0.359131 | 0.094604 | 0.168823 | 0.846924 |
| **T5_alpha** | 0.074492 | 0.359131 | 0.302795 | 0.055359 | 0.678772 | 0.106995 | 0.207764 |
| **T6_alpha** | 0.29333 | 0.638672 | 0.187622 | 0.025574 | 1.0 | 0.359131 | 0.421204 |
| **Fp1beta1** | 0.344869 | 0.890381 | 0.359131 | 0.151428 | 0.678772 | 0.454285 | 0.229309 |
| **Fp2beta1** | 0.015173 | 0.207764 | 0.207764 | 0.120544 | 0.761536 | 0.012451 | 0.008362 |
| **F3_beta1** | 0.031496 | 0.488708 | 0.330261 | 0.229309 | 0.229309 | 0.055359 | 0.012451 |
| **F4_beta1** | 0.105916 | 1.0 | 0.421204 | 0.207764 | 0.524475 | 0.302795 | 0.010254 |
| **F7_beta1** | 0.196786 | 0.187622 | 0.25238 | 0.719727 | 0.761536 | 0.330261 | 0.047913 |
| **F8_beta1** | 0.004145 | 0.207764 | 0.106995 | 0.120544 | 0.719727 | 0.021545 | 6.10E-05 |
| **Fz_beta1** | 0.043596 | 0.846924 | 0.187622 | 0.25238 | 0.187622 | 0.151428 | 0.018066 |
| **C3_beta1** | 0.379454 | 0.890381 | 0.761536 | 0.389404 | 0.934082 | 0.207764 | 0.25238 |
| **C4_beta1** | 0.274717 | 0.678772 | 0.934082 | 0.120544 | 0.599487 | 0.207764 | 0.083252 |
| **Cz_beta1** | 0.036403 | 0.638672 | 0.055359 | 0.761536 | 0.072998 | 0.977966 | 0.008362 |
| **P3_beta1** | 0.196786 | 0.846924 | 0.010254 | 0.934082 | 0.135376 | 0.890381 | 0.047913 |
| **P4_beta1** | 0.274717 | 0.761536 | 0.359131 | 0.421204 | 0.229309 | 0.599487 | 0.094604 |
| **Pz_beta1** | 0.29333 | 0.846924 | 0.25238 | 0.719727 | 0.072998 | 0.803955 | 0.120544 |
| **O1_beta1** | 0.085801 | 0.488708 | 0.072998 | 0.25238 | 0.135376 | 0.977966 | 0.00116 |
| **O2_beta1** | 0.29333 | 0.276855 | 0.276855 | 0.207764 | 0.106995 | 0.846924 | 0.021545 |
| **T3_beta1** | 0.404125 | 0.488708 | 0.229309 | 0.678772 | 0.761536 | 0.389404 | 0.302795 |
| **T4_beta1** | 0.501124 | 0.488708 | 0.135376 | 0.638672 | 0.207764 | 0.524475 | 0.106995 |
| **T5_beta1** | 0.135094 | 0.977966 | 0.04126 | 0.846924 | 0.072998 | 0.846924 | 0.002625 |
| **T6_beta1** | 0.547877 | 0.803955 | 0.599487 | 0.488708 | 0.890381 | 0.846924 | 0.330261 |
| **Fp1beta2** | 0.430127 | 0.599487 | 0.389404 | 0.389404 | 0.934082 | 1.0 | 0.719727 |
| **Fp2beta2** | 0.13984 | 0.846924 | 0.561401 | 0.135376 | 0.421204 | 0.083252 | 0.035339 |
| **F3_beta2** | 0.183895 | 0.599487 | 0.330261 | 0.934082 | 0.135376 | 0.599487 | 0.120544 |
| **F4_beta2** | 0.232773 | 0.389404 | 0.276855 | 0.454285 | 0.047913 | 0.803955 | 0.135376 |
| **F7_beta2** | 0.29333 | 0.803955 | 0.488708 | 0.678772 | 0.719727 | 0.389404 | 0.187622 |
| **F8_beta2** | 0.00296 | 0.454285 | 0.063721 | 0.083252 | 0.488708 | 0.008362 | 0.000305 |
| **Fz_beta2** | 0.171797 | 0.890381 | 0.25238 | 0.488708 | 0.083252 | 0.359131 | 0.561401 |

| | | | | | | | |
|---|---|---|---|---|---|---|---|
| **C3_beta2** | 0.14981 | 0.803955 | 0.072998 | 0.389404 | 0.106995 | 0.207764 | 0.229309 |
| **C4_beta2** | 0.391625 | 0.719727 | 0.359131 | 0.389404 | 0.524475 | 1.0 | 0.187622 |
| **Cz_beta2** | 0.171797 | 0.638672 | 0.229309 | 0.890381 | 0.035339 | 0.168823 | 1.0 |
| **P3_beta2** | 0.379454 | 0.761536 | 0.330261 | 0.276855 | 0.276855 | 0.359131 | 0.678772 |
| **P4_beta2** | 0.948376 | 0.977966 | 0.890381 | 0.561401 | 1.0 | 0.977966 | 0.25238 |
| **Pz_beta2** | 0.839878 | 1.0 | 0.678772 | 0.803955 | 0.678772 | 0.890381 | 0.488708 |
| **O1_beta2** | 0.443637 | 0.719727 | 0.761536 | 0.072998 | 0.719727 | 0.524475 | 0.561401 |
| **O2_beta2** | 0.632497 | 0.389404 | 0.389404 | 0.25238 | 0.803955 | 0.846924 | 0.488708 |
| **T3_beta2** | 0.020345 | 0.106995 | 0.063721 | 0.561401 | 0.276855 | 0.229309 | 0.025574 |
| **T4_beta2** | 0.155043 | 0.454285 | 0.035339 | 0.719727 | 0.359131 | 0.761536 | 0.047913 |
| **T5_beta2** | 0.705535 | 0.599487 | 0.524475 | 0.25238 | 0.359131 | 0.890381 | 0.187622 |
| **T6_beta2** | 0.430127 | 0.524475 | 0.561401 | 0.207764 | 0.524475 | 0.977966 | 0.454285 |
| **Fp1beta3** | 0.45749 | 0.934082 | 0.454285 | 0.421204 | 0.389404 | 0.719727 | 1.0 |
| **Fp2beta3** | 0.043596 | 0.421204 | 0.389404 | 0.229309 | 0.421204 | 0.04126 | 0.018066 |
| **F3_beta3** | 0.183895 | 0.030151 | 0.168823 | 0.803955 | 0.561401 | 0.006714 | 0.035339 |
| **F4_beta3** | 0.430127 | 0.330261 | 0.207764 | 0.719727 | 0.934082 | 0.135376 | 0.168823 |
| **F7_beta3** | 0.614935 | 0.389404 | 0.389404 | 0.524475 | 0.454285 | 0.488708 | 0.302795 |
| **F8_beta3** | 0.006246 | 0.025574 | 0.04126 | 0.187622 | 0.846924 | 0.021545 | 0.006714 |
| **Fz_beta3** | 0.303053 | 0.047913 | 0.168823 | 0.803955 | 0.761536 | 0.207764 | 0.168823 |
| **C3_beta3** | 0.098754 | 0.302795 | 0.168823 | 0.934082 | 0.719727 | 0.055359 | 0.005371 |
| **C4_beta3** | 0.240662 | 0.135376 | 0.229309 | 0.934082 | 0.168823 | 0.599487 | 0.072998 |
| **Cz_beta3** | 0.001072 | 0.094604 | 0.000183 | 0.359131 | 0.021545 | 0.638672 | 0.002625 |
| **P3_beta3** | 0.004996 | 0.524475 | 0.001526 | 0.454285 | 0.002625 | 0.890381 | 0.005371 |
| **P4_beta3** | 0.079954 | 0.055359 | 0.030151 | 0.803955 | 0.599487 | 0.803955 | 0.151428 |
| **Pz_beta3** | 0.003438 | 0.04126 | 0.00116 | 0.561401 | 0.063721 | 0.761536 | 0.135376 |
| **O1_beta3** | 0.032658 | 0.207764 | 0.025574 | 0.678772 | 0.229309 | 0.106995 | 0.002625 |
| **O2_beta3** | 0.29333 | 0.638672 | 0.389404 | 0.488708 | 0.761536 | 0.302795 | 0.025574 |
| **T3_beta3** | 0.877898 | 0.890381 | 0.599487 | 0.421204 | 0.454285 | 0.561401 | 0.421204 |
| **T4_beta3** | 0.564146 | 0.359131 | 0.638672 | 0.488708 | 0.761536 | 0.803955 | 0.330261 |
| **T5_beta3** | 0.013097 | 0.678772 | 0.120544 | 0.890381 | 0.008362 | 0.599487 | 0.004272 |
| **T6_beta3** | 0.102275 | 0.047913 | 0.187622 | 0.599487 | 0.803955 | 0.359131 | 0.04126 |

**Table S4.** The p values for all bands at all lobes during the experi-ments for the 2h-zero calorie intake group.

| Band | Label | Friedman *p* Value | wilcox12 *p* Value | wilcox13 *p* Value | wilcox14 *p* Value | wilcox23 *p* Value | wilcox24 *p* Value | wilcox34 *p* Value |
|---|---|---|---|---|---|---|---|---|
| **delta** | Left.Frontal | 0.258395 | 0.463135 | 0.295776 | 0.714844 | 0.135254 | 0.625732 | 0.241211 |
| **delta** | Right.Frontal | 0.633762 | 0.583008 | 0.241211 | 0.325806 | 0.267578 | 0.714844 | 0.855225 |
| **delta** | Frontal | 0.692196 | 0.951538 | 0.295776 | 0.463135 | 0.135254 | 0.6698 | 0.463135 |
| **delta** | Left.Temporal | 0.068343 | 0.42627 | 0.049438 | 0.903198 | 0.002319 | 0.42627 | 0.013428 |
| **delta** | Right.Temporal | 0.092522 | 0.42627 | 0.390991 | 0.390991 | 0.008545 | 0.029541 | 0.583008 |
| **delta** | Temporal | 0.015054 | 0.501587 | 0.04187 | 0.6698 | 0.003052 | 0.067627 | 0.04187 |
| **delta** | Central | 0.155805 | 0.295776 | 0.104004 | 0.951538 | 0.024536 | 0.501587 | 0.090576 |
| **delta** | Parietal | 0.092522 | 0.541626 | 0.104004 | 0.760864 | 0.013428 | 0.118896 | 0.049438 |
| **delta** | Occipital | 0.063328 | 0.6698 | 0.010742 | 0.6698 | 0.004028 | 0.193726 | 0.029541 |
| **delta** | Whole.Band | 0.027096 | 0.6698 | 0.029541 | 0.501587 | 0.006714 | 0.325806 | 0.135254 |
| **theta** | Left.Frontal | 0.50979 | 0.760864 | 0.625732 | 0.541626 | 0.216553 | 0.807739 | 0.625732 |
| **theta** | Right.Frontal | 0.44756 | 0.067627 | 0.855225 | 0.357544 | 0.357544 | 0.501587 | 1.0 |
| **theta** | Frontal | 0.835756 | 0.295776 | 0.760864 | 0.241211 | 0.357544 | 0.855225 | 0.855225 |
| **theta** | Left.Temporal | 0.048434 | 0.216553 | 0.390991 | 0.760864 | 0.049438 | 0.118896 | 0.216553 |
| **theta** | Right.Temporal | 0.054345 | 0.241211 | 0.067627 | 0.625732 | 0.013428 | 0.501587 | 0.008545 |

| | | | | | | | | |
|---|---|---|---|---|---|---|---|---|
| **theta** | Temporal | 0.046606 | 0.216553 | 0.193726 | 0.714844 | 0.013428 | 0.390991 | 0.024536 |
| **theta** | Central | 0.054345 | 0.078491 | 0.951538 | 0.118896 | 0.049438 | 0.6698 | 0.067627 |
| **theta** | Parietal | 0.054345 | 0.153076 | 0.625732 | 0.193726 | 0.013428 | 0.807739 | 0.049438 |
| **theta** | Occipital | 0.085801 | 0.807739 | 0.216553 | 0.501587 | 0.135254 | 0.541626 | 0.016602 |
| **theta** | Whole.Band | 0.240662 | 0.267578 | 0.501587 | 0.357544 | 0.035278 | 0.714844 | 0.135254 |
| **alpha** | Left.Frontal | 0.341722 | 0.625732 | 0.42627 | 0.760864 | 0.135254 | 1.0 | 0.104004 |
| **alpha** | Right.Frontal | 0.297462 | 1.0 | 0.153076 | 0.951538 | 0.216553 | 0.903198 | 0.118896 |
| **alpha** | Frontal | 0.543291 | 0.903198 | 0.193726 | 0.807739 | 0.135254 | 1.0 | 0.153076 |
| **alpha** | Left.Temporal | 0.258395 | 1.0 | 0.135254 | 0.903198 | 0.010742 | 0.714844 | 0.024536 |
| **alpha** | Right.Temporal | 0.240662 | 0.6698 | 0.057983 | 1.0 | 0.010742 | 0.193726 | 0.067627 |
| **alpha** | Temporal | 0.258395 | 0.760864 | 0.104004 | 1.0 | 0.010742 | 0.295776 | 0.035278 |
| **alpha** | Central | 0.365942 | 0.357544 | 0.390991 | 0.295776 | 0.04187 | 0.583008 | 0.024536 |
| **alpha** | Parietal | 0.308022 | 0.390991 | 0.193726 | 0.463135 | 0.013428 | 0.501587 | 0.029541 |
| **alpha** | Occipital | 0.063328 | 0.903198 | 0.016602 | 1.0 | 0.029541 | 0.541626 | 0.006714 |
| **alpha** | Whole.Band | 0.155805 | 0.625732 | 0.090576 | 0.501587 | 0.024536 | 0.541626 | 0.016602 |
| **beta1** | Left.Frontal | 0.391625 | 0.172607 | 0.6698 | 0.390991 | 0.295776 | 0.501587 | 1.0 |
| **beta1** | Right.Frontal | 0.596462 | 0.463135 | 0.807739 | 1.0 | 0.216553 | 0.625732 | 1.0 |
| **beta1** | Frontal | 0.652929 | 0.193726 | 0.855225 | 0.760864 | 0.295776 | 0.42627 | 0.903198 |
| **beta1** | Left.Temporal | 0.773627 | 0.357544 | 0.463135 | 0.6698 | 0.951538 | 0.807739 | 0.855225 |
| **beta1** | Right.Temporal | 0.224049 | 0.029541 | 0.6698 | 0.325806 | 0.714844 | 0.951538 | 0.42627 |
| **beta1** | Temporal | 0.44756 | 0.118896 | 0.501587 | 0.216553 | 0.903198 | 0.903198 | 0.625732 |
| **beta1** | Central | 0.896432 | 0.295776 | 0.760864 | 0.903198 | 0.760864 | 0.501587 | 0.6698 |
| **beta1** | Parietal | 0.418818 | 0.216553 | 0.153076 | 0.807739 | 0.6698 | 0.501587 | 0.035278 |
| **beta1** | Occipital | 0.330145 | 0.153076 | 0.104004 | 0.583008 | 0.541626 | 0.583008 | 0.104004 |
| **beta1** | Whole.Band | 0.692196 | 0.078491 | 0.42627 | 0.463135 | 1.0 | 0.501587 | 0.807739 |
| **beta2** | Left.Frontal | 0.732528 | 0.583008 | 0.42627 | 0.714844 | 0.357544 | 0.583008 | 0.714844 |
| **beta2** | Right.Frontal | 0.240662 | 0.463135 | 0.625732 | 0.951538 | 0.135254 | 0.267578 | 0.463135 |
| **beta2** | Frontal | 0.652929 | 0.807739 | 0.463135 | 0.855225 | 0.241211 | 0.463135 | 0.625732 |
| **beta2** | Left.Temporal | 0.773627 | 0.541626 | 0.541626 | 0.625732 | 0.501587 | 0.501587 | 1.0 |
| **beta2** | Right.Temporal | 0.258395 | 0.008545 | 0.583008 | 0.295776 | 0.135254 | 0.118896 | 0.855225 |
| **beta2** | Temporal | 0.099748 | 0.04187 | 0.390991 | 0.241211 | 0.267578 | 0.118896 | 1.0 |
| **beta2** | Central | 0.418818 | 0.357544 | 0.903198 | 0.501587 | 0.951538 | 0.241211 | 0.295776 |
| **beta2** | Parietal | 0.835756 | 0.325806 | 0.357544 | 0.807739 | 0.583008 | 0.903198 | 0.118896 |
| **beta2** | Occipital | 0.063328 | 0.035278 | 0.016602 | 0.267578 | 0.583008 | 0.463135 | 0.357544 |
| **beta2** | Whole.Band | 0.18036 | 0.172607 | 0.6698 | 0.855225 | 0.463135 | 0.104004 | 0.625732 |
| **beta3** | Left.Frontal | 0.543291 | 0.390991 | 0.903198 | 0.714844 | 0.501587 | 0.583008 | 0.951538 |
| **beta3** | Right.Frontal | 0.560632 | 0.193726 | 0.325806 | 1.0 | 0.390991 | 0.172607 | 0.463135 |
| **beta3** | Frontal | 0.633762 | 0.118896 | 0.541626 | 0.951538 | 0.583008 | 0.295776 | 0.583008 |
| **beta3** | Left.Temporal | 0.543291 | 0.118896 | 0.295776 | 0.760864 | 0.714844 | 0.541626 | 1.0 |
| **beta3** | Right.Temporal | 0.240662 | 0.057983 | 0.463135 | 0.172607 | 0.501587 | 0.501587 | 0.903198 |
| **beta3** | Temporal | 0.44756 | 0.049438 | 0.42627 | 0.390991 | 0.6698 | 0.501587 | 0.951538 |
| **beta3** | Central | 0.50979 | 0.241211 | 0.118896 | 0.357544 | 0.760864 | 0.951538 | 0.118896 |
| **beta3** | Parietal | 0.896432 | 0.625732 | 0.193726 | 0.583008 | 0.193726 | 0.541626 | 0.295776 |
| **beta3** | Occipital | 0.073742 | 0.172607 | 0.003052 | 0.216553 | 0.153076 | 0.903198 | 0.016602 |
| **beta3** | Whole.Band | 0.232219 | 0.067627 | 0.295776 | 0.714844 | 0.714844 | 0.325806 | 0.216553 |

**Table S5.** The p values for all bands at all lobes during the experi-ments for the non-zero calorie group.

| Band | Label | Friedman | wilcox12 | wilcox13 | wilcox14 | wilcox23 | wilcox24 | wilcox34 |
|---|---|---|---|---|---|---|---|---|
| | | *p* Value | *p* Value | *p* Value | *p* Value | *p* Value | *p* Value | *p* Value |
| **delta** | Left.Frontal | 0.356081 | 0.890381 | 0.454285 | 0.638672 | 0.846924 | 0.761536 | 0.187622 |
| **delta** | Right.Frontal | 0.155043 | 0.934082 | 0.561401 | 0.168823 | 0.330261 | 0.330261 | 0.035339 |
| **delta** | Frontal | 0.232773 | 0.977966 | 0.421204 | 0.135376 | 0.599487 | 0.934082 | 0.083252 |
| **delta** | Left.Temporal | 0.17775 | 0.719727 | 0.135376 | 0.524475 | 0.072998 | 0.276855 | 0.561401 |
| **delta** | Right.Temporal | 0.257177 | 0.890381 | 0.302795 | 0.488708 | 0.094604 | 0.135376 | 0.846924 |
| **delta** | Temporal | 0.265816 | 0.846924 | 0.187622 | 0.454285 | 0.055359 | 0.168823 | 0.846924 |
| **delta** | Central | 0.225123 | 0.524475 | 0.168823 | 0.389404 | 0.063721 | 0.120544 | 0.359131 |
| **delta** | Parietal | 0.232773 | 0.719727 | 0.330261 | 0.135376 | 0.151428 | 0.055359 | 0.421204 |
| **delta** | Occipital | 0.130501 | 0.761536 | 0.04126 | 0.047913 | 0.25238 | 0.207764 | 0.638672 |
| **delta** | Whole.Band | 0.356081 | 0.719727 | 0.151428 | 0.846924 | 0.151428 | 0.454285 | 0.330261 |
| **theta** | Left.Frontal | 0.344869 | 0.934082 | 0.187622 | 0.055359 | 1.0 | 0.488708 | 0.488708 |
| **theta** | Right.Frontal | 0.58075 | 0.599487 | 0.302795 | 0.063721 | 0.934082 | 0.330261 | 0.524475 |
| **theta** | Frontal | 0.344869 | 0.934082 | 0.187622 | 0.055359 | 0.977966 | 0.488708 | 0.359131 |
| **theta** | Left.Temporal | 0.686894 | 0.561401 | 0.803955 | 0.25238 | 0.359131 | 0.678772 | 0.421204 |
| **theta** | Right.Temporal | 0.060184 | 0.561401 | 0.015076 | 0.072998 | 0.330261 | 0.302795 | 0.599487 |
| **theta** | Temporal | 0.705535 | 0.638672 | 0.120544 | 0.276855 | 0.934082 | 0.678772 | 0.890381 |
| **theta** | Central | 0.762613 | 0.599487 | 0.207764 | 0.359131 | 0.890381 | 1.0 | 0.803955 |
| **theta** | Parietal | 0.801252 | 0.890381 | 0.302795 | 0.151428 | 0.846924 | 0.488708 | 0.977966 |
| **theta** | Occipital | 0.313063 | 0.638672 | 0.276855 | 0.063721 | 0.276855 | 0.187622 | 0.678772 |
| **theta** | Whole.Band | 0.614935 | 0.890381 | 0.168823 | 0.047913 | 0.678772 | 0.638672 | 0.803955 |
| **alpha** | Left.Frontal | 0.210513 | 1.0 | 0.229309 | 0.094604 | 0.229309 | 0.120544 | 0.803955 |
| **alpha** | Right.Frontal | 0.430127 | 0.599487 | 0.561401 | 0.678772 | 0.302795 | 0.187622 | 0.890381 |
| **alpha** | Frontal | 0.160449 | 0.803955 | 0.25238 | 0.302795 | 0.187622 | 0.151428 | 0.934082 |
| **alpha** | Left.Temporal | 0.027238 | 0.330261 | 0.135376 | 0.035339 | 1.0 | 0.083252 | 0.359131 |
| **alpha** | Right.Temporal | 0.232773 | 0.599487 | 0.207764 | 0.063721 | 0.561401 | 0.207764 | 0.454285 |
| **alpha** | Temporal | 0.085801 | 0.488708 | 0.120544 | 0.047913 | 0.803955 | 0.083252 | 0.454285 |
| **alpha** | Central | 0.077176 | 0.846924 | 0.151428 | 0.106995 | 0.488708 | 0.094604 | 0.846924 |
| **alpha** | Parietal | 0.379454 | 1.0 | 0.524475 | 0.187622 | 0.719727 | 0.229309 | 0.421204 |
| **alpha** | Occipital | 0.064629 | 0.977966 | 0.302795 | 0.010254 | 0.561401 | 0.229309 | 0.524475 |
| **alpha** | Whole.Band | 0.085801 | 0.803955 | 0.229309 | 0.047913 | 0.599487 | 0.151428 | 0.561401 |
| **beta1** | Left.Frontal | 0.257177 | 0.803955 | 1.0 | 0.151428 | 0.934082 | 0.330261 | 0.025574 |
| **beta1** | Right.Frontal | 0.021103 | 0.302795 | 0.25238 | 0.094604 | 0.599487 | 0.021545 | 0.00116 |
| **beta1** | Frontal | 0.048558 | 0.561401 | 0.638672 | 0.055359 | 0.719727 | 0.04126 | 0.012451 |
| **beta1** | Left.Temporal | 0.29333 | 0.890381 | 0.106995 | 0.719727 | 0.359131 | 0.599487 | 0.072998 |
| **beta1** | Right.Temporal | 0.45749 | 0.934082 | 0.187622 | 0.454285 | 0.359131 | 0.561401 | 0.120544 |
| **beta1** | Temporal | 0.171797 | 0.890381 | 0.120544 | 0.561401 | 0.276855 | 0.524475 | 0.055359 |
| **beta1** | Central | 0.265816 | 0.638672 | 0.421204 | 0.302795 | 0.421204 | 0.207764 | 0.094604 |
| **beta1** | Parietal | 0.105916 | 0.803955 | 0.030151 | 0.977966 | 0.120544 | 0.846924 | 0.106995 |
| **beta1** | Occipital | 0.048558 | 0.330261 | 0.106995 | 0.207764 | 0.094604 | 0.934082 | 0.002014 |
| **beta1** | Whole.Band | 0.020345 | 0.890381 | 0.083252 | 0.302795 | 0.276855 | 0.25238 | 0.004272 |
| **beta2** | Left.Frontal | 0.650354 | 1.0 | 0.934082 | 0.761536 | 0.803955 | 0.977966 | 0.524475 |
| **beta2** | Right.Frontal | 0.029291 | 0.638672 | 0.25238 | 0.063721 | 0.330261 | 0.135376 | 0.006714 |
| **beta2** | Frontal | 0.183895 | 0.977966 | 0.561401 | 0.187622 | 0.761536 | 0.421204 | 0.04126 |
| **beta2** | Left.Temporal | 0.056037 | 0.934082 | 0.055359 | 0.421204 | 0.135376 | 0.302795 | 0.030151 |
| **beta2** | Right.Temporal | 0.762613 | 0.977966 | 0.151428 | 0.890381 | 0.187622 | 0.934082 | 0.072998 |
| **beta2** | Temporal | 0.033862 | 0.638672 | 0.047913 | 0.524475 | 0.106995 | 0.389404 | 0.005371 |
| **beta2** | Central | 0.155043 | 0.561401 | 0.330261 | 0.649497 | 0.083252 | 0.302795 | 0.719727 |

| | | | | | | | | |
|---|---|---|---|---|---|---|---|---|
| **beta2** | Parietal | 0.650354 | 0.678772 | 0.561401 | 1.0 | 0.599487 | 0.599487 | 0.330261 |
| **beta2** | Occipital | 0.650354 | 0.719727 | 0.561401 | 0.151428 | 0.761536 | 0.803955 | 0.524475 |
| **beta2** | Whole.Band | 0.313063 | 1.0 | 0.421204 | 0.359131 | 0.276855 | 0.524475 | 0.004272 |
| **beta3** | Left.Frontal | 0.859009 | 0.229309 | 0.890381 | 0.890381 | 0.207764 | 0.389404 | 0.761536 |
| **beta3** | Right.Frontal | 0.052167 | 0.083252 | 0.083252 | 0.276855 | 0.599487 | 0.055359 | 0.006714 |
| **beta3** | Frontal | 0.45749 | 0.151428 | 0.488708 | 0.524475 | 0.803955 | 0.25238 | 0.106995 |
| **beta3** | Left.Temporal | 0.379454 | 0.389404 | 0.890381 | 0.359131 | 1.0 | 0.599487 | 0.083252 |
| **beta3** | Right.Temporal | 0.379454 | 0.063721 | 0.229309 | 0.846924 | 0.678772 | 0.638672 | 0.25238 |
| **beta3** | Temporal | 0.58075 | 0.846924 | 0.846924 | 0.276855 | 0.678772 | 0.638672 | 0.094604 |
| **beta3** | Central | 0.039129 | 0.229309 | 0.055359 | 0.934082 | 0.135376 | 0.359131 | 0.010254 |
| **beta3** | Parietal | 0.010496 | 0.055359 | 0.002014 | 0.638672 | 0.018066 | 0.890381 | 0.072998 |
| **beta3** | Occipital | 0.085801 | 0.389404 | 0.083252 | 0.524475 | 0.389404 | 0.187622 | 0.008362 |
| **beta3** | Whole.Band | 0.105916 | 0.063721 | 0.025574 | 0.803955 | 0.761536 | 0.276855 | 0.030151 |

**Table S6.** Different brain wave patterns are recorded in the brain's central lobe after two hours. Percentage values refer to the percentage of the relative power changes after two hours compared to the background level.

| Frequency Band | Control Group (Diurnal Effect) | Non-Zero Calorie Group (Diurnal and Exposure Effects) | Effect of Exposure | 2 h-Zero Calorie Group (Diurnal and Exposure and Zero Calorie Effects) | Effect of the Zero Calorie on the Exposure Effect |
|---|---|---|---|---|---|
| **Alpha** | increasing trend 0.61 | increasing trend 11.43 | 10.82% increases | decreasing trend 0.58 | 12.01% mitigating effect |
| **Beta1** | increasing trend 9.30 | decreasing trend 6.70 | 16.0% reduction | decreasing trend 0.16 | 6.45% mitigating effect |
| **Beta2** | increasing trend 5.27 | increasing trend 6.30 | 1.02% increases | increasing trend 2.67 | 3.63% mitigating effect |
| **Beta3** | increasing trend 0.91 | decreasing trend 2.30 | 3.21% reduction | decreasing trend 2.22 | 0.09% mitigating effect |
| **Delta** | decreasing trend 10.30 | decreasing trend 11.02 | 0.72% reduction | decreasing trend 4.13 | 6.89% mitigating effect |
| **Theta** | decreasing trend 1.70 | decreasing trend 5.0 | 3.30% reduction | increasing trend 5.16 | 10.17% mitigating effect |

**Table S7.** Different brain wave patterns recorded in the frontal lobe of the brain after two hours. Percentage values refer to the percentage of the relative power changes after two hours compared to the background level.

| Frequency Band | Control Group (Diurnal Effect) | Non-Zero Calorie Group (Diurnal and Exposure Effects) | Effect of Exposure | 2 h-Zero Calorie Group (Diurnal and Exposure and Zero Calorie Effects) | Effect of the Zero Calorie on the Exposure Effect |
|---|---|---|---|---|---|
| **Alpha** | increasing trend 22.23 | increasing trend 15.01 | 7.22% reduction | decreasing trend 0.82 | 15.83% mitigating effect |
| **Beta1** | increasing trend 15.38 | decreasing trend 11.31 | 26.70% reduction | decreasing trend 8.62 | 2.70% mitigating effect |
| **Beta2** | increasing trend 31.91 | decreasing trend 0.83 | 32.74% reduction | decreasing trend 1.55 | 0.72% exacerbating effect |
| **Beta3** | increasing trend 6.12 | decreasing trend 10.71 | 16.83% reduction | increasing trend 5.53 | 16.25% mitigating effect |
| **Delta** | decreasing trend -18.40 | decreasing trend 0.78 | 17.61% increases | decreasing trend 3.39 | 2.60% exacerbating effect |
| **Theta** | increasing trend 27.13 | decreasing trend 6.74 | 33.86% reduction | increasing trend 7.01 | 13.74% mitigating effect |

**Table S8.** Different brain wave patterns recorded in the occipital lobe of the brain after two hours. Percentage values refer to the percentage of the relative power changes after two hours compared to the background level.

| Frequency Band | Control Group (Diurnal Effect) | Non-Zero Calorie Group (Diurnal and Exposure Effects) | Effect of Exposure | 2 h-Zero Calorie Group (Diurnal and Exposure and Zero Calorie Effects) | Effect of the Zero Calorie on the Exposure Effect |
|---|---|---|---|---|---|
| **Alpha** | decreasing trend 11.50 | increasing trend 3.42 | 14.92% increases | increasing trend 6.28 | 2.86% exacerbating effect |
| **Beta1** | increasing trend 24.05 | decreasing trend 0.63 | 24.68% reduction | decreasing trend 4.25 | 3.62% exacerbating effect |
| **Beta2** | increasing trend 15.32 | decreasing trend 2.57 | 17.89% reduction | decreasing trend 14.39 | 11.81% exacerbating effect |
| **Beta3** | increasing trend 9.16 | decreasing trend 3.34 | 12.50% reduction | decreasing trend 17.03 | 13.69% exacerbating effect |
| **Delta** | increasing trend 20.53 | decreasing trend 11.23 | 31.75% reduction | decreasing trend 16.02 | 4.79% exacerbating effect |
| **Theta** | increasing trend 15.67 | decreasing trend 1.18 | 16.85% reduction | increasing trend 8.98 | 10.17% mitigating effect |

**Table S9.** Different brain wave patterns recorded in the Parietal lobe of the brain after two hours. Percentage values refer to the percentage of the relative power changes after two hours compared to the background level.

| Frequency Band | Control Group (Diurnal Effect) | Non-Zero Calorie Group (Diurnal and Exposure Effects) | Effect of Exposure | 2 h-Zero Calorie Group (Diurnal and Exposure and Zero Calorie Effects) | Effect of the Zero Calorie on the Exposure Effect |
|---|---|---|---|---|---|
| **Alpha** | decreasing trend 8.42 | increasing trend 7.89 | 16.31% increases | decreasing trend 2.08 | 9.97% mitigating effect |
| **Beta1** | increasing trend 18.27 | decreasing trend 2.05 | 20.32% reduction | decreasing trend 1.97 | 0.08% mitigating effect |
| **Beta2** | increasing trend 15.82 | increasing trend 5.10 | 10.72% reduction | increasing trend 2.72 | 2.38% mitigating effect |
| **Beta3** | increasing trend 2.37 | increasing trend 5.66 | 3.29% increases | increasing trend 9.37 | 3.70% exacerbating effect |
| **Delta** | increasing trend 6.69 | decreasing trend 16.29 | 22.97% reduction | decreasing trend 9.69 | 6.60% mitigating effect |
| **Theta** | increasing trend 4.35 | decreasing trend 2.66 | 7.0% reduction | increasing trend 11.41 | 14.07% mitigating effect |

**Table S10.** Different brain wave patterns recorded in the temporal lobe of the brain after two hours. Percentage values refer to the percentage of the relative power changes after two hours compared to the background level.

| Frequency Band | Control Group (Diurnal Effect) | Non-Zero Calorie Group (Diurnal and Exposure Effects) | Effect of Exposure | 2 h-Zero Calorie Group (Diurnal and Exposure and Zero Calorie Effects) | Effect of the Zero Calorie on the Exposure Effect |
|---|---|---|---|---|---|
| **Alpha** | decreasing trend 0.69 | increasing trend 14.03 | 14.71% increases | increasing trend 6.99 | 7.03% mitigating effect |
| **Beta1** | increasing trend 26.47 | increasing trend 4.0 | 22.46% reduction | decreasing trend 5.42 | 9.42% mitigating effect |
| **Beta2** | increasing trend 11.86 | decreasing trend 8.38 | 20.24% reduction | decreasing trend 11.0 | 2.62% exacerbating effect |
| **Beta3** | increasing trend 15.63 | decreasing trend 7.52 | 23.15% reduction | decreasing trend 18.43 | 10.91% exacerbating effect |
| **Delta** | decreasing trend 18.31 | decreasing trend 12.17 | 6.14% increases | decreasing trend 5.97 | 6.21% mitigating effect |
| **Theta** | decreasing trend 1.01 | decreasing trend 4.97 | 3.96% reduction | increasing trend 7.42 | 12.39% mitigating effect |

**Standard Operating Procedure (SOP) for EEG Measurement:**

This SOP outlines the procedure for conducting an electroencephalogram (EEG) brain measurement using ear reference electrodes and gel-based electrodes to ensure accurate data collection and participant safety.

1. The participant was seated in a comfortable chair in a quiet room, ensuring they were relaxed to minimize movement and reduce artifacts during the measurement.
2. The participant's scalp and ears were cleaned using skin preparation gel or alcohol wipes to remove oil, dirt, and hair products, ensuring better electrode conductivity.
3. The cleaned areas were allowed to dry before electrode placement.
4. The EEG cap was placed on the participant's head, with electrodes positioned according to the 10-20 international system (e.g., Fp1, Fp2, C3, C4).
5. For individual electrodes, each one was positioned according to the designated scalp locations.
6. Reference electrodes were attached to the participant's earlobes (A1 on the left ear, A2 on the right ear) to establish ear references.
7. Conductive gel was applied under each electrode using a syringe or applicator to ensure proper contact with the skin.
8. For ear reference electrodes, a thin layer of gel was applied to the electrode pads before attaching them to the ears.
9. Gentle pressure was applied to each electrode to ensure even distribution of the gel and proper skin contact.
10. Impedances were checked to ensure they were below 5kΩ. If any electrodes showed high impedance, gel was reapplied, and adjustments were made as needed.
11. A test recording was conducted to verify signal quality. Any noise or artifacts were identified and corrected before proceeding with the full recording.
12. The EEG recording was started, and the participant's real-time EEG signals were monitored throughout the session.
13. The participant's comfort was continuously monitored, and adjustments were made if necessary to maintain signal quality.
14. After the recording was completed, the electrodes were gently removed from the scalp and ears. Any remaining gel was wiped off with a soft towel or baby wipes.
15. The electrodes and EEG cap were cleaned in accordance with manufacturer instructions. Disposable materials were discarded, and the workspace was cleaned.
16. The EEG data was saved with appropriate labels, including the participant ID, date, and experimental conditions.
17. Observations and any technical issues encountered during the measurement were recorded for future reference.

**File S1: The Questionnaire Form**

Age:

Weight:

Sex: male/female

Participant №

Dear participant,

Thank you for agreeing to the interview. Please answer the questions carefully so that the test results are true and accurate. All your personal information (name, last name, age, history of the disease, and so on) will remain fully reserved and confidential. You are invited to a research that investigates the effect of exposure to aerosol particles from cooking on the human Brain. Based on your answers, we will decide if you are eligible to participate in our experiments.

We will not ask for information that will help us understand your identity. You will have the right to stop being interviewed or not to answer a particular question or be experimented. Your interview and experiment results will be kept confidential and can be withdrawn from the database upon request.

All the details about the experiments will be explained to you today if you are selected. This contains information regarding the experiments and the date, time, and location of your experiments. Those who are excluded from the experiments:

1- Are non-healthy people
2- Suffer from a respiratory, cardiovascular, or nervous disease
3- Suffer from mental disease
4- Do smoking or Shisha
5- Are a major alcohol user
6- Are pregnant women
7- Are drug addicts
8- Are stressed people

1. Do you use tobacco (cigarettes, electronic cigarettes, shisha, etc.)? If you do, how frequently and when was the last time?

___

2. Do you drink alcohol? If yes, how frequently and when was the last time?

___

3. Are you or have you been treated for physical and mental illnesses? If yes, when have you been treated, and for how long?

___

4. Do you have pulmonary sensitivity or pulmonary disease (cough, asthma, lung disease, swelling, etc.?

___

5. What is your profession?

___

6. Do you work in kitchens, restaurants, mining zones, automobile garages, at traffic lights, near highways, or places associated with combustion processes producing certain gases and fumes? If yes, can you write it?

___

7. Do you cook?
At home, how often do you cook fried foods? What type of stove do you use: electric, gas, or solid fuel?

___

8. What is your cooking habit? Do you prefer to cook while ventilation is on or off? If so, what level? Low, medium, or high?

___

9. Do you prefer to cook with low heating level that takes longer for the food to cook or at high heating level that takes faster for the food to cook?

___

10. Is your living place near factories, train stations, highways, or places where certain gases are created? Carbon monoxide, carbon dioxide, volatile organic compounds, etc.?

___

11. How many hours, on average, do you spend outdoors and in traffic in a day?

___

12. Do you have a neurological discomfort associated with the nervous system?

___

13. How many hours a day do you sleep? Do you use sleeping pills?

___

14. How do you assess your stress status in life?

___

## File S2: The Consent Form

**Introduction.** You are invited to participate in a research study entitled

**Interaction of Cooking-Generated Aerosols on the Human Nervous System and the Impact of Caloric Restriction Post-Exposure**

You are asked to participate in a research study. This research team consists of several certified researchers, including research assistants and graduate students from Nazarbayev University, who know the experiments and ethics related to this research. You were selected as a possible participant in this study because you are typically exposed to particles during commuting. Your participation in this research study is voluntary. Those subjects who are excluded from the experiments:

1- Are non-healthy people
2- Suffer from respiratory, cardiovascular, or nervous disease
3- Suffer from mental disease
4- Do smoking or Shisha
5- Are a major alcohol user
6- Are pregnant women
7- Are drug addicts
8- Are stressed people

**Procedures.** PM or particulate matter refers to suspended liquid or solid particles in the air. Particles smaller than 100 nm are called Ultrafine particles. Different sources produce ultrafine particles (nanoparticles) during our daily activity. Such sources are available indoors and outdoors, such as cooking, candle burning, barbequing, traffic exhaust, biomass burning, dust storms, power plant combustions, etc. Ultrafine particles (UFPs) can deposit into the lungs and brain, pass the air-blood barrier, and enter the blood circulation. From that point, they can enter the extrapulmonary organs such as the liver, bladder, heart, and brain. This study investigates the impact of exposure to UFPs indoors on human brain activities. The outcome of this study will help neurologists better understand the causes of neurodegenerative diseases. Thus, your involvement in this study is critical and helpful to the people. This study requires human data during exposure to UFPs to fulfill this objective. The selected volunteers will measure their brain activity using an electroencephalogram (EEG) during cooking experiments.
If you volunteer to participate in this study, the researcher will ask you to do the following:

**Experimental Procedures:**

1. Participants will take the first EEG measurements. This step will be done before being exposed to cooking fumes
2. Participants will take second, third, and fourth EEG measurements 60, 90, and 120 minutes after the cooking.

**Non - experimental procedures:**

Research assistants will make calculations using a paired t-test and ANOVA.

In a questionnaire, participants will be asked about their physical and mental health issues, hours of sleep, amount of stress, and bad habits. The questionnaire to assess the suitability of participants as volunteers will take approximately 5 minutes to complete.

R**isks.** The potential risks of participating in this study are:

There are no significant chronic or acute risks for the volunteers from the experiment. In fact, one of our objectives is to determine the risk of being exposed to UFPs during your daily bus rides. Thus, if you are being tested at your regular rides to your home or workplace, there will be no significant risk associated with you.

**Benefits.** Anticipated benefits from this study are adding the potential benefits to the participants. You may benefit from the study by having a free brain scan and compensation of 5000 KZT.

The research results may lead to a better understanding of the health effects of particles during cooking. Researchers and others may be able to find a better way to minimize the health risks associated with exposure to particulate matter during daily activities.

**Compensation.** There will be tangible compensation of 5000KZT.

**Confidentiality & Privacy.** Any information obtained during this study will be confidential to the fullest extent possible. All efforts, within reason, will be made to keep your personal information in your research record confidential, but total confidentiality cannot be guaranteed. Any information obtained concerning this study that identifies you will remain confidential. It will be disclosed only with your permission or as required by law.

Information I put in my report that could identify you will not be published or shared beyond the research team unless we have your permission. Any data from this research which will be shared or published, will be the combined data of all participants and coded by numbers rather than names.

**Voluntary Nature of the Study**. Participation in this study is strictly voluntary, and if a participation agreement is given, it can be withdrawn at any time without prejudice.

**Statement of Consent**.
I,________________________________________________________________________,
Give my voluntary consent to participate in this study.
The researchers clearly explained to me the background information and objectives of the study and what my participation in this study involves.

I understand that my participation in this study is voluntary. I can withdraw my consent at any time and without giving any reasons, and this will not have any negative consequences for me.

I understand that the information collected during this study will be treated confidentially.

Participants number (optional)________________________________

Signature: ______________________ Date: __________________

Researcher:

Signed__________________________ Date______________________
